# A situational analysis of ear and hearing care in South Korea using WHO ear and Hearing Care Situation Analysis tool

**DOI:** 10.3389/fpubh.2023.1215556

**Published:** 2023-09-28

**Authors:** Juhyeong Lee, Chul Young Yoon, Junhun Lee, Tae Hoon Kong, Seung Ha Oh, Young Joon Seo

**Affiliations:** ^1^Research Institute of Hearing Enhancement, Yonsei University Wonju College of Medicine, Wonju, Republic of Korea; ^2^Department of Biostatistics, Yonsei University Wonju College of Medicine, Wonju, Republic of Korea; ^3^Department of Otorhinolaryngology-Head and Neck Surgery, Yonsei University Wonju College of Medicine, Wonju, Republic of Korea; ^4^Department of Otorhinolaryngology-Head and Neck Surgery, Seoul National University College of Medicine, Seoul, Republic of Korea

**Keywords:** WHO, ear and hearing care, Korean, situation, annual report

## Abstract

**Objectives:**

The WHO emphasizes lifelong management of hearing diseases such as hearing loss and advocates for prevention. The Ear and Hearing Care Situation Analysis (EHCSA) tool was designed by the WHO for assessment and quality improvement of state-led management of hearing loss prevention and management programs. The purpose of this study was to use the EHCSA to assess the ear and hearing management program in Korea and to establish goals consistent with best practices for improving policies and services related to ear and hearing care.

**Methods:**

The EHCSA was used as a need assessment of the ear and hearing management services in the country. The EHCSA consists of two sections. Section 1 consists of 41 questions to evaluate health policies and support services. Section 2 consists of 203 questions to evaluate human resources and services of the ear and hearing management sector.

**Results:**

There are an estimated 800,000 people with hearing loss in Korea. Policies such as hearing aid support are in place, and outreach services such as free hearing tests are also being actively conducted. In all medical institutions, ear and hearing management treatment and medication prescriptions could be received without barriers. Workers in the fields of ear and hearing management, such as audiologists, language therapists, special education teachers, and sign language interpreters, are specialized and have well-established guidelines for training.

**Conclusion:**

Overall, the domestic ear and hearing management sector has confirmed that policies and services are well-prepared in comparison with advanced countries such as the United States, Iran, and China. The use of the EHCSA was functional in collecting data on the current state of domestic ear and hearing management policies and services in Korea, can be used for continuous quality improvement and expansion of medical services, and can be used as a reporting mechanism to the WHO.

## 1. Introduction

Hearing is one of the five senses and refers to a mechanical sense that detects the frequency and intensity of sound waves transmitted through a medium, such as air or water. The ear is a sensory organ that causes brain activity, which leads to thoughts and emotions, and it has a proportion equivalent to vision in perceiving the surrounding environment (1). By 2050, ~2.5 billion people are expected to experience hearing loss, a known representative of hearing disorders, and at least 700 million people will need hearing rehabilitation. According to the WHO, as of 2021, more than 1.5 billion people worldwide have experienced hearing loss. Of these, 430 million suffer from severe hearing loss (2). Failure to treat this condition can affect language development, education, employment, mental health, and depression through human relationships. Globally, the failure to properly address hearing loss costs more than $1 trillion each year. This includes the costs associated with healthcare, education, productivity losses, and social interactions. The causes of hearing loss include aging, noise damage, drug side effects, infections, head trauma, and allergies. It is important to mention that most of these causes can be addressed by healthcare-related services (3). The WHO presented a state-led management program called the Prevention of Deficiency and Hearing Loss to emphasize the importance of lifelong management due to the following reasons: it can occur due to various causes at any time, hearing loss is very common, its prevalence rate is increasing rapidly, and lack of social interest on this matter (4).

Accordingly, since 2005, China has been implementing a national project called the Hearing the Future-China National Hearing Care Program, which includes the early diagnosis, prevention, treatment, and rehabilitation of hearing loss. India has also continued to implement a state-led policy called NPPCD since 2008, and Australia is operating a government-led program to manage and supervise hearing screening, diagnosis, hearing loss prevention, and rehabilitation services from newborns to the older adult. The United States is striving to reduce the occurrence of social problems caused by hearing loss through the national “Healthy People 2020” (5).

In Korea, the number of people diagnosed with hearing loss increased from 280,000 in 2012 to 340,000 in 2016, and the treatment of hearing loss affects the increase in medical costs and hospitalization in future (6). For 2 years from 2007, a pilot project for the early diagnosis of neonatal hearing loss by region has been implemented, and newborn hearing screening and confirmation tests for hearing loss are being conducted mainly for low-income families. In addition, as of 2013, an early diagnosis of hearing loss for low-income newborns nationwide is being implemented, with all newborns being tested for congenital metabolic abnormalities and hearing loss. However, these projects are often implemented by municipal governments. As emphasized by the WHO, the government should strategically establish policies considering the adverse consequences associated with the disease. Other countries use the “Ear and Hearing Care Situation Analysis Tool” (EHCSA) distributed by the WHO to identify their Ear and Hearing Care Situations, report them annually, and prepare strategic health policies accordingly. However, Korea is not reporting to the WHO. EHCSA is significant in evaluating the available direct and indirect policies, services, and human resources associated with ear and hearing therapy, and the healthcare systems of the country are helping to promote and maintain ear and hearing management at all levels of the healthcare system (7). The WHO Collaboration Center is an organization designated by the WHO Secretary-General to form part of an international cooperation network established by the WHO to support programs at national, international, regional, and global levels. According to the WHO Policy and Technology Cooperation Strategy, the WHO Collaboration Center also participates in strengthening national resources in terms of information, services, research, and training to support national health development. The WHO Collaboration Center serves as a support for WHO's mission and program goals, development, and strengthening of institutional capabilities in countries and regions, and there are six institutions in the ear and hearing care field. These include the University of Pretoria in the Republic of South Africa, the University of Indonesia, the Ear Science Institute Australia, the Iran University of Medical Sciences, the China Rehabilitation Research Center for Hearing and Speech Impairment (CRRCHSI), and the National Research Center for Audiology and Hearing Rehabilitation in Russia. To prevent deafness and hearing loss, these institutions provide WHO support through the establishment of information on ear and hearing management, support the implementation of EHCSA manuals, and participate in cooperative projects among WHOCC networks related to ear and hearing care (EHC) issues. As such, it is necessary to submit objective data on EHC in Korea, but it is not currently submitted. Therefore, the Research Institute of Hearing Enhancement offers material for submission. Through this study, the goal is to fill EHCSA and identify the current situation of EHC in Korea to establish and improve policies related to medical services and hearing diseases.

## 2. Methods and materials

### 2.1. Data analysis

The Ear and Hearing Care Situation Analysis Tool (EHCSA) is a method for situation analysis developed by the WHO and includes the conditions of situation analysis (7). Situation analysis refers to several methods that managers use to analyze an organization's internal and external environments to understand its capabilities and environments. Representative examples include 5C analysis, SWOT analysis, and Porter's Five Forces analysis, which use the “WHO EHCSA” developed by the WHO to understand the capabilities and environment of the country. Through this, the WHO emphasizes the current status of Ear & Hearing care in each country and the development of sustainable support measures. Data directly reported to the WHO are not accessible, but some countries have prepared outputs or conducted projects based on the EHCSA. The Islamic Republic of Iran produced its output by implementing a situational analysis of the ear and hearing care program using EHCSA; in Bangladesh, an article using situational analysis through EHCSA was published to aid in the understanding of the status of rehabilitation services for the disabled (8). In Germany, the Rehabilitation Service Assessment Tool was developed based on EHCSA to understand the status of rehabilitation services. The EU recognized the importance of hearing management at the national level through the EHCSA and planned to prepare for future hearing health management policies (9). In addition, to generate awareness, the project collected data related to the use of various hearing aids, their effects, and the correlation between factors affecting hearing damage and accompanying diseases through a big data platform called EVOTION. However, no research has been conducted on the EHCSA in Korea.

The “WHO EHCSA” was distributed to collect, evaluate, and report on ear and hearing management policies, services, and human resources and for the assessment of national health systems in the context of ear and hearing management. This can help to understand the dynamics of hearing loss and the systems and policies that support ear and hearing management, including available personnel, infrastructure, and services, and to establish an improved healthcare system that can help with hearing management. The tool has a total of two sections: Section 1 and Section 2 with a total of 41 and 203 questions, respectively. Section 1 provides information on overall health policies and services, such as total population, age distribution, gender distribution, illiteracy rate, Internet penetration rate, life expectancy, fertility rate, vaccination status, deaf patient distribution, infant health policy, youth health policy, and disease management protocol. Section 2 provides detailed information on human resources, such as departments and representatives dealing with ear and hearing care issues in the Ministry of Health and Welfare, ear and hearing care-related institutions, policies, and programs. Questionnaires for assessment are also available (Supplementary material 1).

### 2.2. Data collection process

The emphasis was on collecting indicators in line with the context of the questions in the WHO ear and hearing care situation analysis tool. Information, such as country profiles and holidays, was collected using documents from national institutions, such as WHO statistics, the Ministry of Health and Welfare, and the National Statistical Office. First, the indicators of the National Statistical Portal (KOSIS) serviced by the National Statistical Office were confirmed. In addition, to confirm the specific information, documents, such as project reports and academic publications of national institutions that calculated the indicators, were checked. Finally, if the index was not secured through the search, consultations were conducted with experts.

### 2.3. National distribution data

To answer the questions, open materials distributed by the state and materials from the conference were used. Data from the National Statistical Office, 2021 population and housing census, 2021 future population estimation, 2020 death cause statistics, and 2017 and 2021 birth statistics press releases were used. In the data from the Korea Centers for Disease Control and Prevention, the 2021 tuberculosis patient report status, 2020 HIV/AIDS report status year, and tuberculosis treatment guidelines were used. In the data from the Ministry of Health and Welfare, clinical guidelines for screening genetic testing for newborns, criteria for determining the grade of disablement, disaster crisis management measures for infectious diseases, and the classification of medical institutions were used. In addition, the World Population Review's literacy rate by the country in 2022, the Ministry of Science and ICTs 2021 wireless communication service subscriber statistics, the 2021 Internet usage survey, the Ministry of Education's school health inspection guidelines and integrated education guidebook, the National Health Insurance Service and Health Insurance Review and Assessment Service's health insurance statistics year, and hospital inquiry services were included. To use data from National Health Insurance, approval of the institutional review board is required. This study was approved by the Institutional Review Board of Yonsei University Wonju Severance Christian Hospital (Wonju, South Korea) (CR323326). The National Statistical Portal (KOSIS) provided by the National Statistical Office, Population and Housing Survey, and Disability Statistical Yearbook published by the Korea Disabled Development Institute were mainly used.

The Korean Statistical Information Service (KOSIS) is a statistical service provided by the National Statistical Office for users to find the statistics they want at once by collecting major domestic and international statistics. Currently, it contains more than 1,000 types of state-approved statistics on the economy, society, and environment prepared by more than 300 institutions and provides the latest statistics on international finance, the IMF, the World Bank, and the OECD. Most national distribution data, such as the population and housing census and disability statistical yearbook, are uploaded on the portal (10).

The population and housing general survey is a basic national statistical survey conducted by the National Statistical Office to understand the size and characteristics of all people and houses in Korea and is released annually. It is a national basic statistical survey conducted on people and residences in the national territory at a certain time and period and is used as basic data in various fields, including policymaking, planning, research, and evaluation by national institutions, private companies, academic organizations, and the public. It is used to prepare secondary processing statistics, such as the Ministry of Employment and Labor's labor supply plan, the Ministry of Health and Welfare's premium rate and national pension fiscal estimate, the low birth rate and aging index, and the Ministry of Land, Infrastructure, and Transport's housing supply rate (11). The disability statistical yearbook is a statistical record that shows the situation of the disabled and has been distributed by the Korea Disabled Development Institute since 2015 (12). It is based on the Convention on the Rights of the Disabled and the satisfaction of the needs of the disabled in different stages of their life cycle. Administrative data of major ministries related to the disabled are recorded and reprocessed to get linked with the performance of the policies for the disabled currently in effect in Korea, indirectly assessed by the government to actively implement policies and services for the disabled and used as basic data to institutionalize welfare policies for the disabled. The disability statistics yearbook consists of 11 social indicators, nine health and welfare yearbooks, seven disability statistics, and 13 social security categories. It collects indicators that represent the lives of the disabled, including a survey on household finance and welfare, annual reports on special education, housing, convenience for transportation, and access to information.

The main priority was whether the criteria for determining the data were calculated by national ministries. As most of the data produced by national ministries are targeted at the entire nation and collected at the national level, it can be inferred that these data have been verified in terms of representation and reliability. If there were no data, the data of the academic society or association in the field were used; if there were no data in the institution, the engineer or expert was consulted. The year of the data to be used was set as 2021, as the data from the major national ministries for the year 2022 would be distributed the following year, and the most recent data were used if there were no data for the year 2021.

## 3. Results

### 3.1. Section 1

#### 3.1.1. Population profile

The results of the population profile are shown in Table 1. The data sources for the total population, age distribution, and sex distribution were the 2021 population and housing census data from the National Statistical Office (11). The data sources for the rural-urban distribution were the 2021 Population and Housing Survey by the Ministry of Land, Infrastructure, and Transport and the 2021 housing survey (11, 13). The data source for the literacy rate was the 2022 literacy rate by country of World Population Review (14).

**Table 1:** Questionnaires of Section 1.

**Subject**	**No**.	**Questionnaires**
Population profile	**1**	**Total population (in millions)**
	Answer	Approximately 51 million
	**2**	**Age distribution of population (%)**
	Answer	0–5 years: 3.3%/6–15 years: 8.7% 16–60 years: 64.4%/>60 years: 23.5%
	**3**	**Sex distribution (%)**
	Answer	Both men and women were 50%
	**4**	**Rural-urban distribution (%)**
	Answer	Rural: 8.6%/Urban: 91.4%/Slum dwellers: 12%
	**5**	**Literacy rate (as a % of the total population)**
	Answer	97.9%
Sociopolitical profile	**1**	**Languages**
	Answer	Korean
	**2**	**% of the population using mobile phone services**
	Answer	Smartphones: 95%/mobile phones: 5%
	**3**	**% of the population reached by Internet services**
	Answer	99.9%
Health status indicators	**1**	**Life expectancy at birth**
	Answer	83.9 years old
	**2**	**Annual birth rate**
	Answer	5.2 per 1,000 people
	**3**	**Under-five mortality rate**
	Answer	3.2 per 1,000 live births
	**4**	**% of births taking place in health facilities**
	Answer	99.6%
	**5**	**% of births taking place at home that are attended by skilled birth attendants**
	Answer	–
	**6**	**Are the following vaccines included in the immunization program of the country? (Measles, Meningitis, Mumps, Rubella, Rubella in adolescents)**
	Answer	Yes
	**7**	**Number of cases of multidrug-resistant tuberculosis per year**
	Answer	371
	**8**	**Number of cases of HIV/AIDS per year**
	Answer	818
Hearing Loss	**1**	**Prevalence of disabling hearing loss**
	Answer	5 million people, 10% of the total population
	**2**	**Age distribution of hearing loss**
	Answer	Teenage: 3.9%/20s: 8.1%/30s: 8.6% 40s: 10.6%/50s: 15%/60s or older: 52%
	**3**	**Incidence of congenital or early-onset childhood hearing loss (ECHL)**
	Answer	6 people per 1,000 live births
	**4**	**What are the main causes of disabling hearing loss?**
	Answer	Chronic otitis media: 0.72%, impacted ear wax: 2.4%, low birth weight: 6.7%, meningitis: 0.02%, mumps: 0.02%, noise-induced hearing loss: 0.17%, ear-toxic hearing loss: 0.0003%, rubella: 0.000004% hearing loss in the older adult: 0.56%
Healthcare strategy	**1**	**Does the country have a national strategy for (Maternal health, child health, eye care, disability and rehabilitation, occupational health, school health, care of older adult, communicable diseases, multidrug-resistant tuberculosis, HIV/AIDS, non-communicable diseases, inclusive education)**
	Answer	Yes
	**2**	**Has the country ratified the United Nations Convention on the Rights of Persons with Disabilities**
	Answer	Yes
	**3**	**Are there existing policies on employment of persons with disabilities?**
	Answer	Yes
	**4**	**Are there any other policies that are relevant to ear and hearing care?**
	Answer	Yes
	**5**	**How would you summarize the overall situation regarding the preparedness of your country for development and implementation of ear and hearing care strategies?**
	Answer	There is awareness among policy-makers about the need for ear and hearing care services. The country is ready to develop and implement a national strategic plan, and resources are available for this purpose

#### 3.1.2. Sociopolitical profile

The results of the sociopolitical profile are shown in Table 1. The data source was the 2021 wireless communication service subscriber statistics of the Ministry of Science and ICT (15) and the 2021 Internet usage survey from the Ministry of Science and ICT (16).

#### 3.1.3. Health status indicators

The results of the health status indicators are shown in Table 1. For data sources, the life expectancy at birth is Statistics Korea's 2021 future population estimate (17), the Annual birth rate is 2021 birth statistics from the National Statistical Office (18), and the under-five mortality rate is statistics from the National Statistical Office's 2020 cause of death (19). The data source of birthplace was the National Statistical Office's 2017 birth statistics press release (20). Regarding birthplace, the National Statistical Office has not collected data since 2017; therefore, the presented data are the latest recorded data. The answer to question 6, “Are the following vaccines included in the immunization program of the country?”, is yes. Regarding vaccines, measles, mumps, and rubella are covered by one MMR vaccine in Korea, and the vaccination rate is 97.7% at the age of 2 years, 97.3% at the age of 3 years, and 95.0% at the age of 6 years. In the case of meningitis, inoculation with both types of Hib and PCV vaccines must be applied to prevent it. The vaccination rate of the Hib vaccine is 98.1% for the age of 1 year, 96.2% for the age of 2 years, 96.1% for the age of 3 years, and 95.9% for the age of 6 years. The vaccination rate for the PCV vaccine is 98.1% at the age of 1 year, 97.0% at the age of 2 years, 96.8% at the age of 3 years, and 97.1% at the age of 6 years. In Korea, the data on the rubella vaccination rate for adolescents were not separately collected, so the answer could not be confirmed. The data source was the National Child Vaccination Status of the Korea Centers for Disease Control and Prevention in 2021 (21). The sources for multidrug-resistant tuberculosis and HIV/AIDS data are the Korea Centers for Disease Control and Prevention's 2021 tuberculosis patient report status (22) and the Korea Centers for Disease Control and Prevention's 2020 Annual Report on HIV/AIDS (23).

#### 3.1.4. Hearing loss

The results of the hearing loss are shown in Table 1. The data source of prevalence and age distribution was the 2021 disease statistics from the Health Insurance Review and Assessment Service (24). The data source for the early onset of child hearing loss was the guidelines of the Ministry of Health and Welfare for neonatal hearing screening. Disease statistics were checked for each major cause of hearing loss. Trauma and periodic factors could not be confirmed owing to personal information protection of national data. The low proportion of people suffering from mumps, meningitis, and rubella was possibly due to vaccination, and the data sources for the answers to this question were the 2021 disease statistics by the Health Insurance Review and Assessment Service, 2020 underweight childbirth rate by the Korea Women's Policy Institute, and 2020 total surveillance infectious disease statistics by the Korea Centers for Disease Control and Prevention (24–26).

#### 3.1.5. Healthcare strategy

The results of the healthcare strategies are shown in Table 1. The first question asks whether there is a national strategy related to the field in parentheses, and all of the answers are “Yes.” Maternal health has guidelines for obstetrics and gynecology in the internship training and treatment guidelines of the Korean Medical Association (27). Child health has clinical guidelines for the genetic screening of newborns distributed by the Ministry of Health and Welfare in 2011 (28). Since 2017, the Ministry of Health and Welfare has implemented an eye care project to prevent blindness in the older adult population. The aim of this project, along with the Korea Blindness Prevention Foundation, was to prevent blindness in the older adult and included eye examination, eye opening surgery, and rehabilitation projects for the older adult with low vision. Disability has a standard for determining the grade of disability, which has been announced by the Ministry of Health and Welfare by law since 2015. Occupational health includes medical management guidelines for workers diagnosed with noise-induced hearing loss in 2021, distributed by the Korea Occupational Safety and Health Agency in 2021, and treatment guidelines for epilepsy and lung disease, distributed in 2018 by the Korean Tuberculosis and Respiratory Society (29). School health has been a sample survey project for school health tests since 1971. Currently, the Ministry of Education oversees it and distributes sample survey operation manuals (30). Representatively, care of the older adult includes dementia-screening projects and dementia treatment management support projects implemented by the Ministry of Health and Welfare. Communicable diseases have a standard manual for disaster crisis management for infectious diseases announced as the Framework Act on Disaster and Safety Management and clinical care guidelines for the treatment of patients with COVID-19 newly distributed by the Korea Institute of Health and Medical Research and the Korean Medical Association (31). Multidrug-resistant tuberculosis has a tuberculosis treatment guideline that has been continuously revised by the Korea Centers for Disease Control and Prevention since 2005, and a new section related to multidrug-resistant tuberculosis was added in 2020 (32). HIV/AIDS has 2020 HIV/AIDS management guidelines distributed by the Korea Centers for Disease Control and Prevention, and the Korea AIDS Prevention Association conducts projects, such as nursing support projects, home welfare projects, AIDS counseling centers, and AIDS prevention education and training (33). Non-communicable diseases have cancer treatment guidelines, such as the 2022 hepatocellular carcinoma treatment guidelines, distributed by the Korean Liver Cancer Society and the National Cancer Center, and the Ministry of Health and Welfare supports projects, such as medical expenses for patients with pediatric, adult, and lung cancers. The Ministry of Education distributed a guidebook for the implementation of integrated elementary and secondary school education in 2017 (34). Korea is a member of the United Nations Convention on the Rights of People with Disabilities. The answer to the question “Has the country implemented policies on employment of persons with disabilities?” is “Yes” (35). The Ministry of Employment and Labor implements employment policies for the disabled, including employment incentives, establishment of standard workplaces, assistance with engineering equipment, employment management cost support, employment of severely disabled people, and transition support for the disabled. The answer to the question “Are there any other policies that are relevant to ear and hearing care?” is “Yes.” The Korea Occupational Safety and Health Service has established a hearing preservation program and implemented policies for workers vulnerable to noise-induced hearing loss, and the Korean Academy of Sciences has distributed guidelines for the treatment of middle ear infections in children in 2014 and guidelines for the development of hearing aids and inspection confirmation in 2016. In the last question of Section 1 as a whole, the following “There is awareness among policy-makers about the need for ear and hearing care services. The country is ready to develop and implement a national strategic plan, and resources are available for this purpose.” can be selected as an answer.

### 3.2. Section 2

#### 3.2.1. Existing strategic plans or policies for ear and hearing care

The results of the EHC policies implemented by the Ministry of Health and Welfare and other institutions are shown in Table 2. The answer to the first question corresponds to “Yes,” and the department dealing with it is the Disabled Policy Bureau of the Ministry of Health and Welfare. The representative is the Director of the Disabled Policy Bureau. The answer to the second question is also “Yes.” Representative programs include the school health examination sample survey project conducted by the Ministry of Education and the hearing preservation program conducted by the Occupational Safety and Health Corporation. Since the Ministry of Health and Welfare is not in charge of the above program, the answer to the third question is “No.” The last question was asked as summarized in 2.1, and we chose “There is a government-led committee or appointed coordinator for EHC. There is also a national strategic plan for EHC. Activities and programs are being implemented throughout the country” as an answer. The Ministry of Health and Welfare is not entirely in charge of the EHC program but other institutions are implementing the program.

**Table 2:** Questionnaires of EHC and service delivery.

**Subject**	**No**.	**Questionnaires**
Existing strategic plan or policies for EHC	**1**	**Is there a designated focal point or coordinator for ear and hearing care in the Ministry of Health?**
	Answer	Yes
	**2**	**Is there an EHC national or sub-national strategy, plan, or program?**
	Answer	Yes
	**3**	**Is the plan led by the Ministry of Health?**
	Answer	No
	**4**	**How would you summarize the overall situation regarding the existence and implementation of strategic plans, programs, and policies in the country?**
	Answer	There is a government-led committee or appointed coordinator for EHC. There is also a national strategic plan for EHC. Activities and programs are being implemented throughout the country
Service delivery	**1**	**Does the country or area have a defined package of services for provision of primary healthcare?**
	Answer	Yes
	**2**	**If yes, is EHC integrated with the package?**
	Answer	Yes
Primary level	**1**	**Is there a health facility at primary level providing healthcare services and child care services and raising health awareness?**
	Answer	Multiple facilities
	**2**	**Who are the healthcare providers at the primary level facilities?**
	Answer	General physicians, ENT specialists, audiologists, speech therapists, hearing aid technicians, teachers of the deaf, sign language interpreters, pediatricians, and obstetricians
	**3**	**What is the population (in 1000s) for which the facility caters?**
	Answer	0.05 per 1,000 people
	**4**	**Which of the following EHC services are provided at this level?**
	Answer	Health awareness, tympanoplasty, information, education and communication, mastoid surgery, ear examination through otoscopy, hearing aid fitting, hearing assessment, cochlear implantation, treatment of acute otitis media, hearing and speech therapy, removal of foreign bodies from the ear, educational support for children with hearing loss, removal of earwax, and grommet insertion services
	**5**	**Are outreach services for EHC available at the primary level?**
	Answer	Yes
	**6**	**If a person reports an ear or hearing problem that requires attention, to whom is he or she referred from the primary level of care?**
	Answer	Private facilities, secondary, and tertiary hospitals
Secondary level	**1**	**Is there a health facility at primary level providing healthcare services and child care services and raising health awareness?**
	Answer	Yes
	**2**	**Who are the healthcare providers at the primary level facilities?**
	Answer	Multiple facilities
	**3**	**What is the population (in 1000s) for which the facility caters?**
	Answer	0.008 per 1,000 people
	**4**	**Which of the following EHC services are provided at this level?**
	Answer	Other than grommet insertion and educational support for children with hearing loss
	**5**	**Are outreach services for EHC available at the primary level?**
	Answer	Free hearing tests for welfare centers
	**6**	**If a person reports an ear or hearing problem that requires attention, to whom is he or she referred from the secondary level of care?**
	Answer	Tertiary hospitals
Tertiary level	**1**	**Are EHC services available at tertiary level?**
	Answer	Yes
	**2**	**What is the health facility at tertiary level providing EHC services?**
	Answer	General hospitals and advanced general hospitals according to the classification of medical institutions by the Ministry of Health and Welfare and Articles 1, 2, and 3 of the Medical Act
	**3**	**What is the population (in 1000s) for which the facility caters?**
	Answer	0.007 per 1,000 people
	**4**	**Who are the healthcare providers at the tertiary level facilities?**
	Answer	ENT specialists
	**5**	**Which of the following EHC services are provided at this level?**
	Answer	Health awareness, tympanoplasty, information, education and communication, mastoid surgery, ear examination through otoscopy, hearing aid fitting, hearing assessment, cochlear implantation, treatment of acute otitis media, hearing and speech therapy, removal of foreign bodies from the ear, and removal of earwax
	**6**	**Are outreach services for EHC available at the tertiary level?**
	Answer	Medical support projects
	**7**	**If a person reports an ear or hearing problem that requires attention, to whom is he or she referred from the tertiary level of care?**
	Answer	Tertiary hospital
	**8**	**What percentage of EHC services and healthcare services are provided by the public health system/ the private sector**
	Answer	71%
	**9**	**How would you summarize the overall situation regarding services for provision of EHC in the country?**
	Answer	EHC services are available at all tertiary, secondary, and primary-level health facilities

#### 3.2.2. Service delivery

The results of the existence of primary medical care and the presence or absence of EHC are shown in Table 2. As primary care also includes otolaryngology, the answer to both questions is “Yes,” and the data source was the article defined for primary care (36).

##### 3.2.2.1. Primary level

The results of the overall state of the primary medical institutions are shown in Table 2. The answer to the first question is “multiple facilities.” There are a total of five primary medical institutions according to the classification of medical institutions by the Ministry of Health and Welfare, and the items are clinics, health centers, health branches, health clinics, and healthcare centers (37). Healthcare providers at primary-level facilities include general physicians, ENT specialists, audiologists, speech therapists, hearing aid technicians, teachers of the deaf, sign language interpreters, pediatricians, and obstetricians. As of 2020, the number of facilities was 2,665, with 2,569 clinics, 91 health centers, and four healthcare centers nationwide, accounting for a ratio of 0.05 per 1,000 people. The data sources were the National Health Insurance Corporation's 2020 regional statistical year of medical use and the current status of lawmakers by city, county, district, and marked subjects (37, 38). At this level, health awareness, tympanoplasty, information, education and communication, mastoid surgery, ear examination through otoscopy, hearing aid fitting, hearing assessment, cochlear implantation, treatment of acute otitis media, hearing and speech therapy, removal of foreign bodies from the ear, educational support for children with hearing loss, removal of earwax, and grommet insertion services are all provided. The data source was the hospital inquiry service of the Health Insurance Review and Assessment Service. The answer to whether primary medical institutions provide outreach services is “Yes.” Many hearing aid companies provide free hearing tests, hearing aid checks, and hearing counseling for the older adult at welfare centers. At the primary level, to learn about hearing problems, patients can visit private facilities, secondary, and tertiary hospitals.

##### 3.2.2.2. Secondary level

The results of the overall state of the secondary medical institutions are shown in Table 2. The answers to the first and second questions are “Yes” and “Multiple facilities,” respectively. Secondary medical institutions include hospitals and nursing hospitals according to the classification of medical institutions by the Ministry of Health and Welfare and Articles 1, 2, and 3 of the Medical Act. In secondary medical institutions, only ENT specialists provide EHC services. As of 2020, there were 241 hospitals and 157 nursing hospitals, with a total of 398 facilities per 1,000 people with a ratio of 0.008. The data sources were the National Health Insurance Corporation's 2020 regional statistical year of medical use and the current status of lawmakers by city, county, district, and marked subjects (37, 38). The EHC services included items other than grommet insertion and educational support for children with hearing loss. Outreach services provide free hearing tests for welfare centers at hearing aid companies, and others. At the secondary level, on learning about hearing problems, patients can visit a tertiary hospital.

##### 3.2.2.3. Tertiary level

The results of the overall state of the tertiary medical institutions are shown in Table 2. The answer to the first question is “Yes.” Tertiary medical institutions include general hospitals and advanced general hospitals according to the classification of medical institutions by the Ministry of Health and Welfare and Articles 1, 2, and 3 of the Medical Act. Only ENT specialists provide EHC services in tertiary medical institutions. As of 2020, there were 187 general hospitals and 42 advanced general hospitals, for a total of 361, and the number of facilities was in the ratio of 0.007 per 1,000 people. The data source was the National Health Insurance Corporation's 2020 Annual Report on Medical Use by Region (38). Medical institutions provide health awareness, tympanoplasty, information, education and communication, mastoid surgery, ear examination through otoscopy, hearing aid fitting, hearing assessment, cochlear implantation, treatment of acute otitis media, hearing and speech therapy, removal of foreign bodies from the ear, and removal of earwax. Municipal governments support outreach services when conducting medical support projects, and the data source was a medical support project for the homeless in Seoul. At the tertiary level, on learning about hearing problems, patients can visit a tertiary hospital. Advanced general hospitals were divided into national and private hospitals, with 12 out of 42 national and public hospitals accounting for 29% and 30 private hospitals accounting for 71%. The last question, “EHC services are available at all tertiary, secondary, and primary level health facilities” can be selected as a summary answer for 2.2.

#### 3.2.3. Health workforce

##### 3.2.3.1. ENT specialists

Section 2.3 concerns people employed in the EHC field. The results of the overall state of ENT specialists are shown in Table 3. As of the fourth quarter of 2020, the number of ENT specialists in Korea was 3,977, with a ratio of 0.8 per 100,000 people. The data source was the current status of the specialist workforce by type of nursing institution 2020 in the National Statistical Portal (39). The minimum education period is 2 years for preparatory courses, 4 years for the main course, 1 year for training, and 4 years for specialists; the source was the Korea Medical University Association. Some institutions educate ENT specialists in Korea, with a total of 40 universities and medical graduate schools. The number of graduates produced annually is 116, based on those who passed the 2020 professional qualification exam. The skills of the ENT specialist include medical management of common ear conditions, surgical management of common ear conditions, myringotomy, grommet insertion, tympanoplasty, mastoid surgery, stapedectomy, cochlear implantation, audiometry, tympanometry, otoacoustic emissions/auditory brainstem response/auditory steady-state response, hearing aid fitting, earmold preparation, audio-verbal therapy, and speech therapy.

**Table 3:** Questionnaires of Health Workforce.

**Subject**	**No**.	**Questionnaires**
ENT specialists	**1**	**Total number in the country**
	Answer	3,977 (0.8 per 100,000 people)
	**2**	**Minimum educational qualification required**
	Answer	2 years for preparatory courses, 4 years for the main course, 1 year for training, and 4 years for specialists
	**3**	**Are there educational institutions offering degree or diploma courses for training of ENT specialists?**
	Answer	Yes
	**4**	**Total number of educational facilities offering degree or diploma in ENT specialists**
	Answer	40 universities and medical graduate schools
	**5**	**Number of persons graduating each year**
	Answer	116
	**6**	**Skills of the ENT specialists**
	Answer	Medical management of common ear conditions, surgical management of common ear conditions, myringotomy, grommet insertion, tympanoplasty, mastoid surgery, stapedectomy, cochlear implantation, audiometry, tympanometry, otoacoustic emissions/auditory brainstem response/auditory steady-state response, hearing aid fitting, earmold preparation, audio-verbal therapy, and speech therapy
Audiologists	**1**	**Total number in the country**
	Answer	475 (0.5 per 100,000 people)
	**2**	**Minimum educational qualification required**
	Answer	4 years
	**3**	**Are there educational institutions offering degree or diploma courses for training of audiologists?**
	Answer	Yes
	**4**	**Total number of educational facilities offering degree or diploma in audiology**
	Answer	6 universities in Korea based on the Department of Language and Hearing.
	**5**	**Number of persons graduating each year**
	Answer	210
	**6**	**Skills of the audiologists**
	Answer	Audiometry, tympanometry, otoacoustic emissions/auditory brainstem response/auditory state response, hearing aid fitting, audio-verbal therapy, speech therapy, family counseling, counseling on the use of hearing aids, earmold preparation, use of environmental aids, otoscopy, and diagnosis of common ear conditions
Speech therapists and Audio-verbal therapists	**1**	**Total number in the country**
	Answer	2,800
	**2**	**Minimum educational qualification required**
	Answer	4 years
	**3**	**Are there educational institutions offering degree or diploma courses for training speech therapists or audio-verbal/auditory-oral therapists?**
	Answer	Yes
	**4**	**Total number of educational facilities offering degree or diploma course**
	Answer	29 universities in Korea with language therapy-related departments
	**5**	**Number of persons graduating each year**
	Answer	900
	**6**	**Skills of teachers of the speech therapists and Audio-verbal therapists**
	Answer	Audio-verbal therapy, speech therapy, audiometry, and counseling on the use of environmental aids
Hearing aid and earmold technicians	**1**	**Total number in the country**
	Answer	288
	**2**	**Minimum educational qualification required**
	Answer	2 years
	**3**	**Are there educational institutions offering degree or diploma courses for training of Hearing aid or earmold technicians?**
	Answer	Yes
	**4**	**Total number of educational facilities offering degree or diploma**
	Answer	6 universities related to audiology
	**5**	**Number of persons graduating each year**
	Answer	260
	**6**	**Skills of the hearing aid or earmold technicians**
	Answer	Audiometry, tympanometry, family counseling, speech therapy, audio-verbal therapy, and counseling on the use of environmental aids
Teachers of the deaf	**1**	**Total number in the country**
	Answer	650 (1.2 per 100,000 people)
	**2**	**Minimum educational qualification required**
	Answer	4 years
	**3**	**Are there educational institutions offering degree or diploma courses for training of teachers of the deaf?**
	Answer	Yes
	**4**	**Total number of educational facilities offering degree or diploma**
	Answer	36 universities based in the Department of General Education and Special Education
	**5**	**Number of persons graduating each year**
	Answer	180
	**6**	**Skills of teachers of the deaf**
	Answer	Educational support for preschool children with hearing loss, educational support for school children with hearing loss, sign language practice, family counseling, and counseling on the use of hearing aids and environmental aids
Sign language interpreters	**1**	**Total number in the country**
	Answer	2,968 (5.7 per 100,000 people)
	**2**	**Minimum educational qualification required**
	Answer	1 year, and an additional 50 h of maintenance training must be completed after obtaining a sign language interpreter certificate
	**3**	**Are there educational institutions offering degree or diploma courses for training of sign language interpreters?**
	Answer	Yes
	**4**	**Total number of educational facilities offering degree or diploma**
	Answer	4 departments including the Korean Deaf Association and Sign Language Interpretation-related departments
	**5**	**Number of persons graduating each year**
	Answer	100
	**6**	**Skills of sign language interpreters**
	Answer	Sign language interpretation, family and individual counseling, and counseling on the use of hearing aids and environmental aids
General physicians	**1**	**Total number in the country**
	Answer	6,030 (11.6 per 100,000 people)
	**2**	**Minimum educational qualification required**
	Answer	6 years
	**3**	**Which of the following ear and hearing care services do general physicians provide?**
	Answer	Diagnosis and medical management of acute ear infections, diagnosis and medical management of chronic ear infections, hearing tests, and removal of ear wax and foreign bodies
Emergency medical technician	**1**	**Total number in the country**
	Answer	11,000 (22 per 100,000 people)
	**2**	**Minimum educational qualification required**
	Answer	6 months
	**3**	**Which agency provides training for emergency medical technicians?**
	Answer	Firefighting schools and Emergency rescue education centers
	**4**	**Which of the following ear and hearing care services do emergency medical technicians provide?**
	Answer	Raising health awareness in the community, medical management of ear infections, reference services, and removal of foreign bodies
Health education specialist	**1**	**Total number in the country**
	Answer	14,000 (28 per 100,000 people)
	**2**	**Minimum educational qualification required**
	Answer	4 years
	**3**	**Which agency provides training for health education specialists?**
	Answer	The Korea Health Association, the Korea Nursing Association, the International Association for Moderation, and the Korea Health Promotion and Development Institute
	**4**	**Which of the following ear and hearing care services do emergency health education specialists provide?**
	Answer	Raising health awareness in the community, medical management of early influences, and reference services according to the notification of national qualification management for health educators
Caregivers	**1**	**Total number in the country**
	Answer	13,000 (26 per 100,000 people)
	**2**	**Minimum educational qualification required**
	Answer	6 months
	**3**	**Which agency provides training for caregivers?**
	Answer	Caregiver center
	**4**	**Which of the following ear and hearing care services do caregivers provide?**
	Answer	Raising health awareness in the community, medical management of early influences, referenced services, wax, and foreign body removal
Practical nurse	**1**	**Total number in the country**
	Answer	400,000 (800 per 100,000 people)
	**2**	**Minimum educational qualification required**
	Answer	1 year
	**3**	**Which agency provides training for practical nurses?**
	Answer	Practical nurse training centers and Practical nurse academies
	**4**	**Which of the following ear and hearing care services do practical nurses provide?**
	Answer	Raising health awareness in the community, medical management of early influences, referenced services, wax, and foreign body removal
Summarize	**1**	**How would you summarize the overall situation regarding availability of human resources for ear and hearing care within the country?**
	Answer	There are an adequate number of human resources for EHC available in all urban and rural areas of the country
	**2**	**How would you summarize the overall situation regarding availability of educational facilities for training of human resources for EHC within the country?**
	Answer	Training facilities for health workers are available as well as educational facilities for professional training, and these are adequate to provide EHC for the entire country

##### 3.2.3.2. Audiologists

The results of the overall state of audiologists are shown in Table 3. As of 2022, the number of audiologists in Korea is 475, with a ratio of 0.5 per 100,000 people. The data source was the audiologist's qualification examination center. The minimum training period is 4 years. There are educational instruments and six universities in Korea based on the Department of Language and Hearing. The number of audiologists produced annually is 210, and the data source was the audiologist qualification examination center. Audiologists' skills include audiometry, tympanometry, otoacoustic emissions/auditory brainstem response/auditory state response, hearing aid fitting, audio-verbal therapy, speech therapy, family counseling, counseling on the use of hearing aids, earmold preparation, use of environmental aids, otoscopy, and diagnosis of common ear conditions.

##### 3.2.3.3. Speech and audio-verbal therapists

The results of the overall state of speech and audio-verbal therapists are shown in Table 3. As of 2022, there are 2,800 language rehabilitation workers in Korea, and the data source was the Korean Language Rehabilitation Association. The minimum education period for speech and audio-verbal therapists is 4 years. There are educational instruments and 29 universities in Korea with language therapy-related departments. The number of speech and audio-verbal therapists produced annually is ~900, and the source was the current status of applicants for the language rehabilitation certificate of the National Institute of Health (40). Skills of speech and audio-verbal therapists include audio-verbal therapy, speech therapy, audiometry, and counseling on the use of environmental aids.

##### 3.2.3.4. Hearing aid and earmold technicians

The results of the overall state of hearing aid and earmold technicians are shown in Table 3. As of 2022, there are 288 hearing aid distributors in Korea, the data source of which was the Korean Hearing Society. Hearing aid technicians and earmold technicians have a minimum educational period of 2 years, and there are educational instruments and six universities related to audiology in Korea. The number of hearing aid and earmold technicians produced annually is ~260, and the data source was the status of those who had passed the hearing management certificate of the Korean Acoustics Association (41). The skills of hearing aid and earmold technicians include audiometry, tympanometry, family counseling, speech therapy, audio-verbal therapy, and counseling on the use of environmental aids.

##### 3.2.3.5. Teachers of the deaf

The results of the overall state of teachers of the deaf are shown in Table 3. As of 2020, the number of teachers in this department in Korea was 650, with a ratio of 1.2 per 100,000 people. The data source was the 2020 Special Education Statistics of the Ministry of Education. The minimum training period is 4 years. There are educational institutions and 36 universities based in the Department of General Education and Special Education. The annual number of teachers in the department was about 180 in 2020, and the data source was the Ministry of Education's 2020 special education statistics. The skills of teachers of the deaf include educational support for preschool children with hearing loss, educational support for school children with hearing loss, sign language practice, family counseling, counseling on the use of hearing aids and environmental aids, and the data source was the Korean Occupational Dictionary of the Ministry of Employment and Labor (42).

##### 3.2.3.6. Sign language interpreters

The results of the overall state of sign language interpreters are shown in Table 3. As of 2018, the number of sign language interpreters in Korea was 2,968, with a ratio of 5.7 per 100,000 people. The source was research data on how to revitalize sign language interpreters provided by the Korea Disabled Development Institute (43). The minimum training period is 1 year, and an additional 50 h of maintenance training must be completed after obtaining a sign language interpreter certificate. There are educational instruments and a total of four departments, including the Korean Deaf Association and Sign Language Interpretation-related departments. According to the Ministry of Education's 2020 Special Education Statistics, the number of sign language interpreters produced annually is ~100. Skills of sign language interpreters include sign language interpretation, family and individual counseling, and counseling on the use of hearing aids and environmental aids.

##### 3.2.3.7. General physicians

The results of the overall state of general physicians are shown in Table 3. As of the fourth quarter of 2020, the number of general physicians in Korea was 6,030, with a ratio of 11.6 per 100,000 people. The data source was the current state of the national statistical portal. The minimum training period is 6 years (39). General physicians can provide diagnosis and medical management of acute ear infections, diagnosis and medical management of chronic ear infections, hearing tests, and removal of ear wax and foreign bodies.

##### 3.2.3.8. Health workers

Health workers, excluding medical personnel (doctors, dentists, oriental doctors, nurses, midwives, pharmacists, and herbalists) specified in the Medical Act, include emergency medical technicians, caregivers, health education specialists, and practical nurses.

###### 3.2.3.8.1. Emergency medical technician

The results of the overall state of emergency medical technicians are shown in Table 3. As of 2020, ~11,000 emergency medical technicians were employed in Korea, with a ratio of 22 per 100,000 people. The source was statistics on the emergency medical status of the National Medical Center (44). The minimum training period is 6 months, according to the emergency rescue test information of the National Testing Service, a Korean healthcare institution. Educational institutions have firefighting schools and emergency rescue education centers according to the Emergency Medical Act (45). Emergency responders can provide services, such as raising health awareness in the community, medical management of ear infections, reference services, and removal of foreign bodies. The data source was the Korean vocational dictionary of the Ministry of Employment and Labor.

###### 3.2.3.8.2. Health education specialist

The results of the overall state of health education specialists are shown in Table 3. Health education specialists are those who provide education related to health and health promotion and specialize in conducting health promotion projects. As of 2021, about 14,000 people were employed in Korea, with a ratio of 28 per 100,000 people. The data source was a survey on the current status of health education personnel activities at the Korea Institute of Health Promotion and Development (46). The minimum education period is 4 years, depending on the matter in the survey on the status of health education history activities of the Korea Health Promotion and Development Institute. According to the Ministry of Health and Welfare's notice on the national qualification management of health educators, there is the Korea Health Association, the Korea Nursing Association, the International Association for Moderation, and the Korea Health Promotion and Development Institute. Health educators can provide services, such as raising health awareness in the community, medical management of early influences, and reference services, according to the notification of national qualification management for health educators.

###### 3.2.3.8.3. Caregiver

The results of the overall state of caregivers are shown in Table 3. As of 2020, ~13,000 caregivers were employed in Korea, with a ratio of 26 per 100,000 people. The data source was the National Health Insurance Corporation's Statistical Yearbook of Long-Term Care Insurance for the older adult (47). The minimum education period is 6 months in accordance with the Ministry of Health and Welfare's 2021 guidelines for training caregivers, and the educational institution is the caregiver center. Caregivers can provide services for raising health awareness in the community, medical management of early influences, referenced services, and wax and foreign body removal, according to the guidelines of the Ministry of Health and Welfare (48).

###### 3.2.3.8.4. Practical nurse

The results of the overall state of practical nurses are shown in Table 3. As of 2020, ~400,000 practical nurses were employed in Korea, with a ratio of 800 per 100,000 people. The data source was the 2020 Health and Medical Personnel Survey of the Ministry of Health and Welfare (49). The minimum training period is 1 year, depending on the qualification for applying for the practical nurse examination at the National Examination Institute, a Korean healthcare institution. Educational institutions include practical nurse training centers and practical nurse academies, according to the rules of nursing assistants and medical-related businesses of the Ministry of Health and Welfare (50). Practical nurses can provide services by raising health awareness in the community, medical management of early influences, referenced services, and wax and foreign body removal, according to the 2021 guidelines of the Ministry of Health and Welfare for nursing care education.

##### 3.2.3.9. Summarize

Table 3 comprehensively summarizes about domestic medical workers, and the summarized answer to the first question can be selected as “There are an adequate number of human resources for EHC available in all urban and rural areas of the country.” The summarized answer to the second question can be selected as “Training facilities for health workers are available as well as educational facilities for professional training, and these are adequate to provide EHC for the entire country.”

#### 3.2.4. Medical products and health technology

##### 3.2.4.1. Hearing devices

###### 3.2.4.1.1. Hearing aids

The results of the overall state of hearing aids are shown in Table 4. The answer to the first question is “Yes,” and digital technology, analog technology, body-worn, behind-the-ear, in-the-ear, open-fit, and custom-made earmolds are all possible. For the third and fourth questions, there is a hearing aid fitting guidance for each hearing aid company, but it is not provided separately by grade and child age. Hearing aid is covered under insurance, so the answer to the fifth question is “at full cost.” Regarding the maintenance of hearing aids, there are guidelines for each company, so the answer is “Yes,” and the fitting program manual of Resound, one of the hearing aid companies, was checked (51). Battery provision is not included in the public hearing aid fitting program, and hearing aid services can be received in the private sector. Representative institutions among non-governmental organizations include Snail of Love and Dasan.

**Table 4:** Questionnaires of Medical products and health technology.

**Subject**	**No**.	**Questionnaires**
Hearing aids	**1**	**Are hearing aid services available through the public health system?**
	Answer	Yes
	**2**	**What types of hearing aids are available through the public health system?**
	Answer	Digital technology, analog technology, body-worn, behind-the-ear, in-the-ear, open-fit, and custom-made earmolds are all possible
	**3**	**Is there standardized guidance or recommendations for hearing aid fitting in adults?**
	Answer	Hearing aid fitting guidance for each hearing aid company
	**4**	**Is there standardized guidance or recommendations for hearing aid fitting in children?**
	Answer	Hearing aid fitting guidance for each hearing aid company
	**5**	**Are hearing aids fitted:** **□Free of cost** **□at subsidized cost** **□at full cost**
	Answer	At full cost
	**6**	**Are repair and maintenance issues included in the hearing aid fitting program?**
	Answer	Yes
	**7**	**Is battery provision part of the hearing aid fitting program?**
	Answer	No
	**8**	**Are hearing aid services available in the private sector?**
	Answer	Yes
	**9**	**Are any non-governmental organizations providing hearing aid services?**
	Answer	Snail of Love and Dasan
Cochlear implants	**1**	**Are CI services available through the public health system?**
	Answer	Yes
	**2**	**Are CI services available in the private sector?**
	Answer	Yes
	**3**	**Is there standardized guidance or recommendations on cochlear implantation?**
	Answer	The guidelines are publicly distributed by Snail of Love, a non-profit organization
Other assistive devices	**1**	**Are loop systems available in public places?**
	Answer	Yes
	**2**	**Are loop systems available in schools or special schools?**
	Answer	Yes
	**3**	**Are captioning services available on majority of TV channels?**
	Answer	Yes
Medicines	**1**	**Which of the following broad-spectrum antibiotics are available through the public health system and at which care level?**
	Answer	All
	**2**	**Which of the following antibiotic ear drops are available through the public health system and at which care level?**
	Answer	All
	**3**	**Which of the following antifungal ear drops are available through the public health system and at which care level?**
	Answer	All
	**4**	**Which of the following nasal decongestants are available through the public health system and at which care level?**
	Answer	All
	**5**	**How would you summarize the overall situation regarding the availability and accessibility of hearing devices in the country?**
	Answer	Hearing aid services are available and accessible to most people in urban and rural areas of the country. CI are available and accessible to those in need of them

###### 3.2.4.1.2. Cochlear implants

Cochlear implants are available in both public health systems and the private sector, similar to hearing aids. This was confirmed in the hospital inquiry service of the Health Insurance Review and Assessment Service, and the guidelines are publicly distributed by Snail of Love, a non-profit organization (52).

##### 3.2.4.2. Other assistive devices

The results of the overall state of other assistive devices are shown in Table 4. All the answers to questions about loop systems are “Yes,” and Wave Hearing carried out installments at Incheon International Airport, a church in Gangnam, Seoul, Hyundae Motor Studio in Goyang, Daejeon's Cathedral, and Cheonan's Independence Hall. In addition, installments were carried out in several elementary and middle schools by applying the hearing-loop system to the Goyang Special Education Support Office. Captioning services are also available on major TV channels. The guidelines for the provision of broadcasting programs for the disabled announced by the Korea Communications Commission stipulate that “closed caption broadcasting, screen commentary broadcasting, and Korean sign language broadcasting should be produced and organized in terrestrial broadcasting,” and there were 41 terrestrial broadcasters in 2021 (53).

##### 3.2.4.3. Medicines

The results of the overall state of medicines are shown in Table 4. According to the Prescription Act, Article 17 of the Medical Act, broad-spectrum antibiotics, antibiotic ear drops, antifungal ear drops, and nasal decongestants can be prescribed at all medical institutions because no classification of drugs can be selectively prescribed according to the medical institution. The answer to the last question is a summary of 2.4, and since measures and guidelines are in place for all questions, “Hearing aid services are available and accessible to most people in urban and rural areas of the country. CI are available and accessible to those in need of them” (54).

#### 3.2.5. Health financing

##### 3.2.5.1. Financing of ear and hearing care services

The results of the overall state of enactment of the hearing care service are shown in Table 5. The answer to the first question is “Yes.” The Ministry of Health and Welfare had set up a budget for 2021 in the name of training public medical personnel, strengthening the publicity of local hospitals, and training professionals in vulnerable areas (55). The government is conducting projects, such as the newborn hearing loss test support project and hearing aid support project, but the support rate differs depending on income, so the appropriate answer selection would be “Some services are available free of charge, but others have to be paid for, fully or partially” (56). In Korea, some of the medical expenses are covered by health insurance, so we choose “Costs are partially covered by health insurance” as the answer. For the last question, “EHC services are completely free” would be a reasonable choice.

**Table 5:** Questionnaires of Health financing and Health information and research.

**Subject**	**No**.	**Questionnaires**
Financing of ear and hearing care services	**1**	**Is there a budget allocation for EHC in the Ministry of Health?**
	Answer	Yes
	**2**	**Does the government provide EHC services?**
	Answer	Yes
	**3**	**Are EHC services provided by privately practicing specialists?**
	Answer	Yes
	**4**	**Are EHC services provided by any non-governmental agency?**
	Answer	Yes
Health insurance	**1**	**Is health insurance available in the country?**
	Answer	Yes
	**2**	**If yes, what percentage of the population is covered by it?**
	Answer	99%
	**3**	**Are EHC services provided by privately practicing specialists?**
	Answer	Yes
	**4**	**Who are the key providers of health insurance in the country?**
	Answer	Government
Health information system	**1**	**Does the country have a national health information system?**
	Answer	Yes
	**2**	**Are health-related data and information collected and centrally administered in the country?**
	Answer	Yes
	**3**	**What are the sources of health information reported by the country?**
	Answer	Program reports
	**4**	**Is there a system of personal child health records (through health or other departments)?**
	Answer	Yes
	**5**	**Is information on ear or hearing included as part of this record?**
	Answer	Yes
	**6**	**Is any information or indicator related to EHC reported at government level?**
	Answer	Yes
Research	**1**	**Are there any government-led agencies or institutes conducting research in the field of EHC?**
	Answer	Yes
	**2**	**What are the key areas of focus of the research in the field of EHC?**
	Answer	Clinical
	**3**	**Which are the key funding agencies supporting research in the field of EHC?**
	Answer	The Korea Research Foundation and the Korea Academic Promotion Foundation
	**4**	**How would you summarize the overall situation regarding the health information system in the country?**
	Answer	There is a government-led health information system in the country. It includes some information or indicators on EHC

##### 3.2.5.2. Health insurance

The results of the overall state of health insurance are shown in Table 5. The answer to the first question is “Yes.” On 1 November 2020, out of the total population of about 51.829 million living in Korea, 51.345 million people subscribed to the National Health Insurance, accounting for 99% of the population. The data source was the 2020 health insurance statistical yearbook published by the National Health Insurance Service and the Health Insurance Review and Assessment Service (57). Compared to the 78% private health insurance subscription rate in Korea as of 2020, the key provider of health insurance in the country was the government. The data source was the 2020 private medical insurance subscription rate distributed by the National Statistics Portal (58). The answer to the last question is about the summary of 2.5, and “Most of the cost of EHC is covered through government-led health financing schemes, and it is affordable for all.” is the appropriate answer.

#### 3.2.6. Health information and research

##### 3.2.6.1. Health information system

The results of the overall state of health-related data are shown in Table 5. The answers to the first and second questions are “Yes” because the National Health Insurance Service (NHIS), the Health Insurance Review and Assessment Service (HIRA), the National Cancer Center, and the Korea Centers for Disease Control and Prevention exist. In the case of the National Health Insurance Service and the Health Insurance Review and Assessment Service, the answer to the third question is “Program reports” because it is reported based on the information of the subjects subscribed to the national health insurance. The answers to the fourth and fifth questions are “Yes” because there is an infant screening cohort database among the databases of the National Health Insurance Service and early or hearing information is included in the DB. Although there are no data that the government reports only EHC distribution, the answer to the last question is “Yes” because there are data, such as the annual disability statistics, released by the Korea Disabled Development Institute (12).

##### 3.2.6.2. Research

The results of the overall state of EHC research are shown in Table 5. The first question is whether there is a government-led institution that conducts EHC research in Korea. The answer is “Yes” because the Korea Vocational Ability Research Institute is conducting research in the field of hearing management as a learning module development and utilization project. The second question asked about the focus of domestic EHC research, and referring to an article listed in the Korean academic journal Index in otolaryngology in 2022, we answered with “Clinical” because there were a maximum number of clinical papers. The third question is whether any institutions support EHC research; this is the case with the Korea Research Foundation and the Korea Academic Promotion Foundation. The last answer for the summary question of 2.6. is “There is a government-led health information system in the country. It includes some information or indicators on EHC.” since EHC research is being actively conducted in Korea and the government is also supporting it.

## 4. Discussion

This study aimed to identify the current status of EHC in Korea using EHCSA distributed by the WHO in 2015, compare it with other countries, and establish and improve policies related to medical services and hearing diseases. To this end, we would like to examine various areas, such as domestic health policies, the status of patients with hearing-impairment, and the status of medical personnel, and report to WHO on the state of EHC in Korea.

In Iran, a situation analysis was conducted on the domestic ear and hearing care program based on the EHCSA. Through this, inadequate health literacy, weak intra-sectoral and intersectoral cooperation, inadequacy of policy responses, non-integration of the EHC in the primary healthcare system, poor standard processes and resources of EHC, and lack of an EHC surveillance system were identified as major problems (8). In Bangladesh, ~1,200 experts were interviewed using the EHCSA, with insufficient rehabilitation staff training, lack of qualified medical personnel, insufficient primary and secondary medical facilities, and high rates of care service charges (59). Twenty-two countries in sub-Saharan Africa had less than one ENT specialist, audiologist, and speech therapist per 100,000 people in all countries except South Africa (60). In some countries, such as Burundi and Malawi, there are no cultivated speech therapists (61). In Guatemala, ENT specialists were present in a ratio of 2.8 per 1 million people and 61 per 1 million people in Argentina, while in Southeast Asia, ENT specialists were in a ratio of 2.68 per 100,000 people in all countries except Thailand, and audiologists, speech experts, and sign language interpreters were all in low numbers (62). The WHO used the WHO Workforce Indicator for Staffing Needs tool to assess the human resource gap in EHC. The gap in education for the development of related service experts was most prominent in low- and middle-income countries. In the case of arrogance, all those affected by earwax need 137 ENT specialists to be treated, with only 40 being able to treat, showing a 70% availability rate. In India, 1,075 ENT specialists are required to identify and diagnose common ear diseases in all patients up to the age of 15 years (63). At a tertiary medical institution in Santiago, Chile, 78 experts fit hearing aids, with ~0.01% of them in charge of the work (64).

In contrast, Russia supplemented its nationwide implant and hearing aid programs by introducing a cochlear implant in 1991. Therefore, infants born with hearing impairments could receive hearing aids or cochlear implants and rehabilitation services as needed, and up to 1,100 infants receive cochlear implants each year. In addition, people of all ages can receive hearing tests and hearing aid fitting services at audiological centers, which are paid through the national budget (65). In the UK, the National Health Service (NHS) supports people in need of hearing aids and cochlear implants. Approximately 750,000 hearing aids and cochlear implants are provided through the NHS each year, and a national supply chain of hearing aids has been established to provide them at low prices (66). In China, various measures are being implemented to bridge the gap in human resources, such as auditory and verbal therapy. Since 1995, the Specialized Educational Program has been launched in collaboration with hearing and language rehabilitation experts, and more than 1,000 experts have been trained to date, providing hearing and language rehabilitation and aiming to establish a Chinese rehabilitation university. In addition, vocational training courses have been conducted since 2008 for high-quality hearing aid fitting (67).

Comparing policies and workforce status in other countries, data, such as Korea's EHC program and workforce status, are at the level of advanced countries. The Ministry of Health and Welfare publishes a health and welfare white paper every year to understand major policies and achievements in the health and welfare sector and to help the government achieve new national goals and tasks. The policies implemented by the Ministry of Health and Welfare, the current status of projects, and plans for implementing new policies are specified. There are child welfare policies, older adult welfare policies, low birth rate measures, disability welfare policies, oral health policies, mental health policies, and herbal medicine policies; however, EHC policies are not implemented or planned, except for innate hearing tests and hearing aid support policies (68).

The Korea Health Industry Promotion Agency's “Hearing Aid Market Analysis” published in 2015 reported that the average growth rate of hearing aids worldwide is 6.9%. Looking at the ranking, Korea showed the fourth-highest growth rate of 8.5% after 18.3% in China, 10.3% in Germany, and 9.1% in France (69). There are ~12,250 audiologists, 5,570 hearing aid sellers working as hearing experts, and ~13,000 hearing aid centers in the United States. Japan has 3,750 hearing aid centers, and Korea has 1,582 hearing aid centers that have been reported and are operating (70).

A survey was conducted to investigate the current status of the domestic hearing aid market, and 61.6 and 71.7% of the respondents said they needed an obscure certificate to sell hearing aids in the category of factors that hinder the development of the domestic hearing aid market and need education as a hearing expert, respectively (71). In addition, it is difficult to determine the exact status of the supply of hearing aids because anyone can sell them by reporting to the medical device sales business (72).

The above data are not updated continuously, so it is difficult to state that it represents the current situation because it is not the latest data and the survey was conducted with a limited number of people. Therefore, this study is meaningful, and the information on the latest status of the EHC programs was collected. Overall, both the Health workforce and EHC programs are well-prepared, but incidences of hearing diseases, such as hearing loss, are continuously increasing, so various support measures need to be prepared to convert hearing aid-related certificates into national certificates or rehabilitation and support. In addition, domestic health policy improvements and medical service expansion should be achieved through the continuous collection of data on EHC.

## Data availability statement

The original contributions presented in the study are included in the article/Supplementary material, further inquiries can be directed to the corresponding author.

## Ethics statement

The studies involving humans were approved by the Institutional Review Board of Yonsei University Wonju Severance Christian Hospital (CR323326). The studies were conducted in accordance with the local legislation and institutional requirements. Written informed consent for participation was not required from the participants or the participants' legal guardians/next of kin in accordance with the national legislation and institutional requirements.

## Author contributions

Conceptualization: YS. Data curation: JuhL and JunL. Formal analysis, methodology, and visualization: CY and JuhL. Project administration, writing—reviewing, and editing: CY, YS, SO, and TK. Writing—original draft: JuhL. All authors contributed to the article and approved the submitted version.
