# Supplementary material for: A situational analysis of ear and hearing care in South Korea using WHO ear and Hearing Care Situation Analysis tool
Source: Front Public Health. 2023 Sep 28;11:1215556. doi: 10.3389/fpubh.2023.1215556 (PMC10569215; doi:10.3389/fpubh.2023.1215556)
Supplement: Supplementary file 1 [file Data_Sheet_1.PDF]

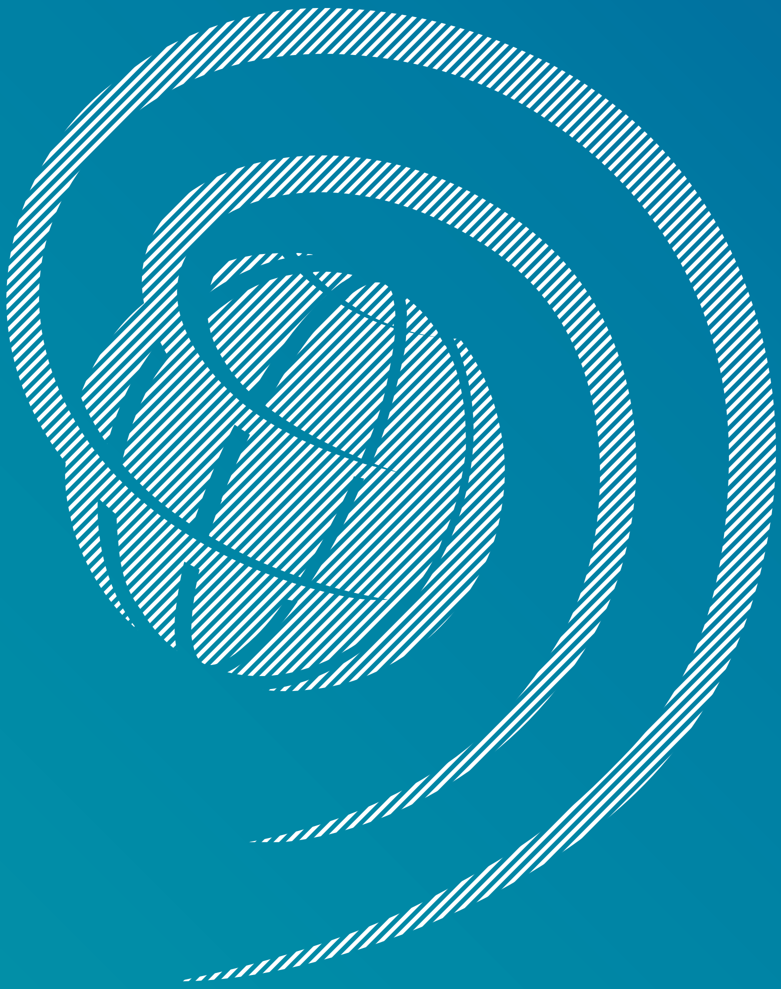

EAR AND HEARING CARE

SITUATION ANALYSIS TOOL

World Health Organization

The cover image has been developed in collaboration with the IDA Institute and the China Research and Rehabilitation Centre for Deaf Children, Beijing China.

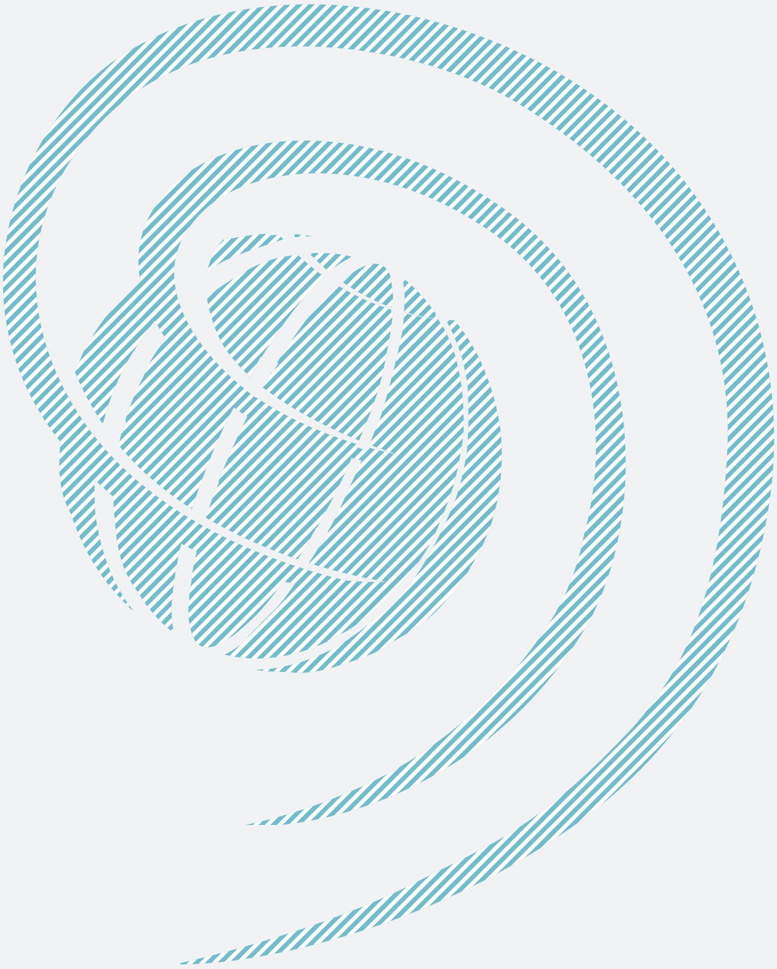

EAR AND HEARING CARE

SITUATION ANALYSIS TOOL

World Health Organization

WHO Library Cataloguing-in-Publication Data:

Ear and hearing care: situation analysis tool.

1.Hearing Loss. 2.Hearing. 3.Ear Diseases. 4.Health Surveys. I.World Health Organization.

ISBN 978 92 4 150995 4 (NLM classification: WV 270)

© World Health Organization 2015

All rights reserved. Publications of the World Health Organization are available on the WHO website (www.who.int) or can be purchased from WHO Press, World Health Organization, 20 Avenue Appia, 1211 Geneva 27, Switzerland (tel.: +41 22 791 3264; fax: +41 22 791 4857; e-mail: bookorders@who.int).

Requests for permission to reproduce or translate WHO publications –whether for sale or for non-commercial distribution– should be addressed to WHO Press through the WHO website (www.who.int/about/licensing/copyright_form/en/index.html).

The designations employed and the presentation of the material in this publication do not imply the expression of any opinion whatsoever on the part of the World Health Organization concerning the legal status of any country, territory, city or area or of its authorities, or concerning the delimitation of its frontiers or boundaries. Dotted and dashed lines on maps represent approximate border lines for which there may not yet be full agreement.

The mention of specific companies or of certain manufacturers' products does not imply that they are endorsed or recommended by the World Health Organization in preference to others of a similar nature that are not mentioned. Errors and omissions excepted, the names of proprietary products are distinguished by initial capital letters.

All reasonable precautions have been taken by the World Health Organization to verify the information contained in this publication. However, the published material is being distributed without warranty of any kind, either expressed or implied. The responsibility for the interpretation and use of the material lies with the reader. In no event shall the World Health Organization be liable for damages arising from its use.

Printed by the WHO Document Production Services, Geneva, Switzerland

CONTENTS

4 Acknowledgements

5 DEVELOPMENT OF THE SITUATION ANALYSIS TOOL

6 BACKGROUND AND PURPOSE

7 USING THE TOOL

8 OUTLINE OF THE TOOL

9 QUESTIONNAIRE

10 Section 1: General Country Information

14 Section 2: Assessment of health system capacity

38 Section 3: Stakeholders analysis

48 Section 4: List of sources

50 SUGGESTED REPORTING FORMAT

54 Annex 1: Guidance for use

ACKNOWLEDGEMENTS

This document is the outcome of a consultative process led by the World Health Organization. It has been reviewed by the following WHO staff members: Shelly Chadha, Alarcos Cieza, Alison Harvey and Etienne Krug.

The conceptual foundations of the document were outlined at the WHO Ad hoc Consultation on Promotion of Ear and Hearing Care in Member States, held at WHO Headquarters in Geneva on 1–2 April 2015. Participants in this meeting were: Arun Kumar Agarwal, Mazin Al-Khabori, Pierre Anhoury, José Barajas, Xingkuan Bu, Louise Carroll, Lucy Carter, Charlotte M. Chiong, Michael Chowen, Jackie L. Clark, Suneela Garg, Christian Garms, Khalid Abdul Hadi, Alejandro Hernández, Linda J. Hood, Isaac Macharia, Thierry Mom, Alfred Mwamba, Katrin Neumann, Augusto Peñaranda, Suchitra Prasansuk, Diego Santana, Sandhya Singh, Andrew W. Smith, George Tavartkiladze and Jean Wilson.

The working group for development of this tool consisted of Pierre Anhoury, Steve Crump, Bulantrisna Djelantik, Suneela Garg, Sally Harvest, Diego Santana, Sandhya Singh and Andrew Smith. The tool was field-tested and evaluated by Suneela Garg, Isaac Macharia, Abdel-Moneim Minshawi and Peter Thorne.

DEVELOPMENT OF THE SITUATION ANALYSIS TOOL

This tool is the outcome of a consultative process. The relevance of situation analysis as an important step in the development of a national ear and hearing care strategy was established by the WHO Ad hoc Consultation on Promotion of Ear and Hearing Care in Member States, at a meeting at WHO Headquarters in Geneva on 1–2 April 2015. The outline of the situation analysis tool was agreed during this meeting. On the basis of these discussions and a review of other situation analysis tools, an outline of the structure and contents was developed by WHO. A working group was then set up to review the draft at a meeting on 1–2 July. The revised draft went through two more rounds of revision by the working group, and was also reviewed by the WHO regional offices. It then underwent field evaluation in Egypt, Fiji, India, Kenya and New Zealand. On the basis of the inputs and comments received from the reviewers, the tool was once again modified and reviewed within WHO.

BACKGROUND AND PURPOSE

Millions of people in the world live with disabling hearing loss that could have been prevented or effectively managed through effective ear and hearing care. Many conditions that lead to hearing loss can be prevented, for example otitis media, exposure to excessive noise, trauma, exposure to ototoxic substances, and infections such as meningitis, measles, mumps and rubella. Many people with hearing loss can be supported through early identification and appropriate interventions.

Ear and hearing care refers to comprehensive, evidence-based interventions to prevent, identify and treat ear diseases and hearing loss, and to rehabilitate and support persons with hearing loss. In order to provide such services, countries must have a strategic plan that takes into account the demographic profile, requirements and resources.

The first step in the development of such a strategy is an assessment of the situation in the country regarding ear and hearing care. This step is crucial to an understanding of the epidemiology of hearing loss and the status of the systems supporting ear and hearing care, including the available human resources, infrastructure and services. It allows needs to be assessed and the gap between the current situation and the ideal one to be defined. An understanding of the existing health system and of the programmes that can potentially intersect with hearing care will provide important background for the development of a strategy. Stakeholder analysis is a key component of setting up a collaborative and cohesive work plan.

The situation analysis tool facilitates this first step of planning for ear and hearing care. It is intended to help attain the following objectives:

- to assess the available direct and indirect policies, services and human resources related to ear and hearing care;
- to describe the framework and functioning of the health care system in the country, in the context of ear and hearing care;
- to assess the need for ear and hearing care services;
- to identify opportunities for promoting and sustaining integrated ear and hearing care along the continuum of care,[1] at all levels of the health system.

The situation analysis tool is primarily intended for use at national and subnational levels[2] by ear and hearing care planners, ear and hearing care health professionals and policy-makers, as well as by the international and domestic stakeholders working with them. The tool supports the compilation of information that can be used for advocacy, for development of a new strategy or updating of existing strategic plans for ear and hearing care. It facilitates the review of ear and hearing care services, to highlight the gaps and needs for service provision. The results can inform evidence-based decision-making, ensuring efficient use of available resources.

The information from the situation analysis should be used to plan an ear and hearing care strategy. A separate manual published by WHO[3] provides guidance on this process.

[1] Continuum of care is a concept involving an integrated system of care that guides and tracks patient over time through a comprehensive array of health services spanning all levels of care.

[2] Subnational level refers to a well-defined administrative region of the country, such as a state, canton, district or province.

[3] Ear and hearing care: planning and monitoring of national strategies. A manual. Geneva, World Health Organization, 2015.

USING THE TOOL

A situation analysis may be initiated by the Ministry of Health, ear and hearing care professionals or nongovernmental organizations (NGOs) working in the field. Technical assistance in conducting a situation analysis or using the results is available from WHO. The tool is essentially a questionnaire that can be used to collect the necessary information. The questionnaire itself is given in section 5. Annex 1 provides additional information on the concepts, definitions and methods used, and should be read before the situation analysis is undertaken.

The methods described below may be used for gathering the information for the questionnaire. More tips about possible sources of information are provided in Annex 1.

a. Desk-based data collection

Information such as the country profile, burden of disease, epidemiology of hearing loss, and health status indicators can be obtained from existing peer-reviewed literature, WHO statistics, the national statistics office/board and Ministry of Health documents.

b. Interviews with relevant stakeholders

Stakeholders include:

- Ministry of Health: focal point for hearing care, noncommunicable diseases or disability; focal point for maternal and child health programmes; focal point for tuberculosis; other officials, as required; a list of possible interviewees and their contact details should be made;
- WHO country office staff;
- chairperson or coordinator of the National Committee for Ear and Hearing Care or of subnational committees, where they exist;
- representatives of associations of people who are deaf or hard of hearing, community-based rehabilitation programmes and disability rights organizations;
- key professionals involved in ear and hearing care service provision or planning in the country; such people can be identified through the Ministry of Health or professional organizations;
- members of statutory bodies, such as councils of health professionals;
- officers of professional organizations and societies, e.g. of ear, nose and throat specialists, otologists, audiologists and public health specialists;
- representatives of training institutions;
- local and international NGOs working in the field of ear and hearing care.

The situation analysis tool can be shared when the interview is arranged, and any specific areas to be addressed by the individual highlighted. Interviews may be recorded as hand-written notes or – where permitted and possible – as audio or video recordings.

c. Review and analysis of information

The information collected should be collated and organized in the provided format. Sources of information should be recorded as far as possible. Where documentary evidence is not available, the source or person who provided the information should be included in the list.

d. Summary tables

Summary tables should be completed carefully and used to record the progress of the country on different aspects of ear and hearing care services, over a period of time.

OUTLINE OF THE TOOL

The situation analysis tool questionnaire has the following sections.

SECTION 1: GENERAL COUNTRY INFORMATION:

- Population distribution and profile
- Sociopolitical profile
- Health status indicators
- Hearing loss prevalence
- Health care strategy

SECTION 2: ASSESSMENT OF HEALTH SYSTEM CAPACITY

- **Leadership and governance:** information on national committee and existing plans and programmes for ear and hearing care.
- **Service Delivery:** information on provision of direct and integrated ear and hearing care along the continuum of care.
- **Health workforce:** health care providers (direct and indirect) for ear and hearing care, at all levels of the health care system.
- **Medical products and health technology:** information on diagnostic tests, equipment, hearing and other devices and medicines.
- **Health information and research:** mechanism for recording and reporting health related information.
- **Health financing:** seek information on financing of ear and hearing care services and health insurance.

SECTION 3: STAKEHOLDER ANALYSIS:

potential stakeholders in ear and hearing care and other possible partners.

SECTION 4: LIST OF SOURCES

QUESTIONNAIRE

SECTION 1: GENERAL COUNTRY INFORMATION

**Name of the country and area (state, region, province, district) (if relevant) for which this situation analysis is being undertaken**	
**Name of the person in charge of this situation analysis**	
Designation and qualification	
Institution	
Address	
Email	
Telephone numbers	

1.1	Population profile	
1.1.1	**Total population (in millions)**	
1.1.2	**Age distribution of population (%)**	0 – 5 years: .... %<br>6 – 15 years: .... %<br>16 – 60 years: .... %<br>> 60 years: .... %
1.1.3	**Sex distribution (%)**	Male: .... %<br>Female: .... %
1.1.4	**Rural-urban distribution (%)**	Rural: .... %<br>Urban: .... %<br>Slum[4] population, (if applicable): .... %
1.1.5	**Literacy rate (as a % of total population)**	.... %
1.1.6	**Any other population-related factor that needs to be considered in planning services**	

[4] Urban slums are a "run-down area of a city characterized by substandard housing and squalor and lacking in tenure security. They are residential areas with a lack of durable housing, insufficient living area, lack of access to clean water, inadequate sanitation and insecure tenure." (United Nations Human Settlements Programme (UN–HABITAT). 2006 Annual report. Kenya, 2006.)

1.2	**Sociopolitical profile** (indicate sources)
1.2.1	**Languages:**
1.2.2	**% of population using mobile phone services:** .... %
1.2.3	**% of population/area reached by internet services:** .... %

1.3	**Health status indicators** (include sources of information in section 4)
1.3.1	**Life expectancy at birth:** .... years
1.3.2	**Annual birth rate:** .... per 1000 population
1.3.3	**Under-five mortality rate:** .... per 1000 live births
1.3.4	**% of births taking place in health facilities (institutional deliveries):** .... %
1.3.5	**% of births taking place at home that are attended by skilled birth attendants:** .... %
1.3.6	**Immunization:**<br><br>**Are the following vaccines included in the immunization programme of the country?**<br><br>If yes, give coverage.<br><br>• Measles: ☐ yes ☐ no: .... % coverage<br>• Meningitis: ☐ yes ☐ no: .... % coverage<br>• Mumps: ☐ yes ☐ no: .... % coverage<br>• Rubella: ☐ yes ☐ no: .... % coverage<br>• Rubella in adolescents: ☐ yes ☐ no: .... % coverage<br><br>*Attach detailed immunization schedule*
1.3.7	**Number of cases of multidrug-resistant tuberculosis per year:** ....
1.3.8	**Number of cases of HIV/AIDS per year:** ....

1.4	**Hearing loss** (indicate sources)
1.4.1	**Prevalence of disabling hearing loss:** ....**%**<br>If information on disabling hearing loss is not available, indicate the definition used in estimation of prevalence:<br>..<br>..
1.4.2	**Age distribution of hearing loss (if available)**<br>• 0 – 4 years: .... %<br>• > 4 – 15 years: .... %<br>• > 15 – 60 years: .... %<br>• > 60 years: .... %
1.4.3	**Incidence of congenital or early-onset childhood hearing loss (ECHL):** .... per 1000 live births

1.4.4 What are the main causes of disabling hearing loss (rank in order of prevalence)?

Causes	Ranking (1 – 11)
Chronic otitis media	
Impacted ear wax	
Low birthweight	
Meningitis	
Mumps, measles	
Noise-induced hearing loss	
Ototoxicity	
Perinatal factors	
Presbycusis	
Rubella	
Trauma	

Comments and additional information:

1.5	**Health care strategy**	
1.5.1	**Does the country have a national strategy for:**	
	• maternal health:	☐ yes ☐ no
	• child health:	☐ yes ☐ no
	• eye care:	☐ yes ☐ no
	• disability and rehabilitation:	☐ yes ☐ no
	• occupational health:	☐ yes ☐ no
	• school health:	☐ yes ☐ no
	• care of elderly:	☐ yes ☐ no
	• communicable diseases:	☐ yes ☐ no
	• multidrug-resistant tuberculosis:	☐ yes ☐ no
	• HIV / AIDS:	☐ yes ☐ no
	• noncommunicable diseases:	☐ yes ☐ no
	• inclusive education:	☐ yes ☐ no
1.5.2	**Has the country ratified the United Nations Convention on the Rights of Persons with Disabilities**	☐ yes ☐ no
1.5.3	**Are there existing policies on employment of persons with disabilities?**	☐ yes ☐ no
1.5.4	**Are there any other policies that are relevant to ear and hearing care?**<br>If yes, *please specify and attach*	☐ yes ☐ no
Comments and additional information:		

How would you summarize the overall situation regarding the preparedness of your country for development and implementation of ear and hearing care strategies?

Circle the most suitable response. If none of the statements accurately reflects the situation in your country, add what seems most appropriate in your country's context.

**1**	There is no awareness among policy-makers in the country about the need for ear and hearing care services. Development and implementation of a strategic plan for hearing care is highly unlikely at present.
**2**	There is some awareness among policy-makers about the need for ear and hearing care services. However, development and implementation of a strategic plan for hearing care is unlikely at present.
**3**	There is awareness among policy-makers about the need for ear and hearing care services. A strategic plan for ear and hearing care is likely to be developed soon.
**4**	There is awareness among policy-makers about the need for ear and hearing care services. The country is ready to develop and implement a national strategic plan, provided resources are available.
**5**	There is awareness among policy-makers about the need for ear and hearing care services. The country is ready to develop and implement a national strategic plan, and resources are available for this purpose.
**6**	None of the above. The current status can be stated as:

SECTION 2: ASSESSMENT OF HEALTH SYSTEM CAPACITY

2.1	Leadership and governance
2.1.1	**Focal point and national committee**<br>Is there a designated focal point or coordinator for ear and hearing care in the Ministry of Health?<br>☐ yes ☐ no<br>If yes, provide details<br>Name: ....<br>Address: ....<br>Telephone number: ....<br>Email: ....<br>If no, please go to **2.1.1a**
	**Is there a Ministry of Health-led national committee to address the issue of hearing care?**<br>☐ yes ☐ no<br>If yes: name of committee ....<br>(Attach details of members and terms of reference, if available, as separate sheet.)<br>If no, go straight to **2.1.2**
	**Who chairs the committee?**<br>Name: ....<br>Designation: ....
	**Name and contact details of focal point of the committee**<br>Name: ....<br>Address: ....<br>Telephone number: ....<br>Email: ....
	**What is the frequency of its meetings?**<br>☐ Quarterly<br>☐ Biannual<br>☐ Annual<br>☐ Other (please specify): ....

2.1.1a	**Are there any subnational committees?**<br>☐ yes ☐ no<br><br>If yes, please provide details on a separate sheet.

Comments and additional information:

2.1.2 Existing strategic plan or policies for ear and hearing care (EHC)

Is there an EHC national or sub national strategy, plan or programme?

☐ yes ☐ no ☐ other

If yes or other, outline the details of the strategy on a separate sheet

If no, go to **2.1.3**

Is the plan led by the Ministry of Health?

☐ Yes ☐ No

If no, which agency leads the plan? ....

Comments and additional information:

2.1.3 Hearing care programmes

Is there a government-led infant hearing screening programme?
☐ yes ☐ no

If yes, please attach relevant programme details, including report, if available

Who leads the programme?

Which parts of the country or area are covered by the programme?

What percentage of the population is covered by the programme?

If no, is there a government-led neonatal or infant screening programme for congenital diseases?
☐ yes ☐ no

Is there a government-led school hearing screening programme?
☐ yes ☐ no

If yes,

who leads the programme?

Which parts of the country/area are covered by the programme?

What percentage of the population is covered by the programme?

If no, is there a government-led school health programme?
☐ yes ☐ no

Is there a government led programme for provision of hearing devices?
☐ yes ☐ no

If yes, who leads the programme?

Which hearing devices are provided under this programme?

Which parts of the country/area are covered by the programme?

What % of population is covered by the programme?

If no, is there a government-led programme for provision of other assistive devices?
☐ yes ☐ no

Is EHC integrated into other policies or strategies such as those for:

☐ child health
☐ tuberculosis management
☐ occupational health
☐ strategies for healthy ageing
☐ disability policies
☐ provision of assistive devices
☐ others (please list):

Comments and additional information:

How would you summarize the overall situation regarding the existence and implementation of strategic plans, programmes and policies in the country?

Circle the most suitable response. If none of the statements accurately reflects the situation in your country, add what seems most appropriate in your country's context.

1	There is no government led committee or appointed coordinator for EHC.<br>There is no national strategy or plan and no other programmes that address ear and hearing care.
2	There is a government led committee or appointed coordinator for EHC. There is as yet no national strategy or plan for EHC.
3	There is a government led committee or appointed coordinator for EHC.<br>A national strategic plan has been developed but has not yet been implemented.
4	There is a government led committee or appointed coordinator for EHC.<br>There is a national strategic plan for EHC and activities are being implemented in some parts of the country, but do not cover the entire country.
5	There is a government led committee or appointed coordinator for EHC. There is a national strategic plan for EHC. Activities and programmes are being implemented throughout the country.
6	None of the above. The current status can be stated as:

2.2 Service delivery

2.2.1 **Does the country or area have a defined package of services for provision of primary health care?**

☐ yes ☐ no

If yes, is EHC integrated in the package? ☐ yes ☐ no

2.2.2 Primary level

Is there a health facility at primary level providing health care services and child care services and raising health awareness?

☐ yes ☐ no ☐ multiple facilities

If yes, what type of facility is it? ....

If no, who provides health care at the primary level? ....

Who are the health care providers at the primary level facilities?
(Please tick all service providers who are involved in health care services at primary level facilities.)

- ☐ General physicians
- ☐ ENT specialists
- ☐ Audiologists
- ☐ Speech therapists or audio-verbal therapists
- ☐ Hearing aid technicians or ear mould technicians
- ☐ Teachers of the deaf
- ☐ Sign-language interpreters
- ☐ Paediatricians
- ☐ Obstetricians
- ☐ Others (please specify): ....

What is the population (in 1000s) for which the facility caters? ....

Which of the following EHC services are provided at this level?
(Please tick all services that are available at primary level facilities.)

- ☐ Health awareness
- ☐ Information, education and communication
- ☐ Ear examination through otoscopy
- ☐ Hearing assessment
- ☐ Treatment of acute otitis media
- ☐ Removal of foreign bodies from ear
- ☐ Removal of ear wax
- ☐ Grommet insertion
- ☐ Tympanoplasty
- ☐ Mastoid surgery
- ☐ Hearing aid fitting
- ☐ Cochlear implantation
- ☐ Hearing and speech therapy
- ☐ Educational support for children with hearing loss
- ☐ Others (please specify): ....

Are outreach services for EHC available at the primary level?

☐ yes ☐ no

If yes, please give details about the nature of the services, frequency and coordinating authority on a separate sheet

If a person reports with an ear or hearing problem that requires attention, to where is he or she referred from the primary level of care?

- ☐ Secondary level hospital
- ☐ Tertiary hospital
- ☐ Private facility
- ☐ Other (please specify): ....
- ☐ There is no referral

Comments and additional information:

2.2.3 Secondary level

Are EHC services available at the secondary level?

☐ yes ☐ no

Is there a health facility at secondary level providing EHC services?

☐ yes ☐ no ☐ multiple facilities

If yes, what type of facility is it? ....

If there are more than one type of facility then mention all (like district hospitals and civil hospitals)

If no, which facility provides health care at the secondary level? ....

What is the population (in 1000s) for which the facility caters? ....

Who are the health care providers at the secondary level facilities?

(Please tick all service providers who are involved in health care services at the secondary-level facilities.)

- ☐ General physicians
- ☐ ENT specialists
- ☐ Audiologists
- ☐ Speech therapists or audio-verbal therapists
- ☐ Hearing aid technicians or ear mould technicians
- ☐ Teachers of the deaf
- ☐ Sign-language interpreters
- ☐ Paediatricians
- ☐ Obstetricians
- ☐ Others (please specify): ....

Which of the following EHC services are provided at this level?

(Please tick all services that are available at secondary level facilities.)

- ☐ Health awareness
- ☐ Information, education and communication
- ☐ Ear examination through otoscopy
- ☐ Hearing assessment
- ☐ Treatment of acute otitis media
- ☐ Removal of foreign bodies from ear
- ☐ Removal of ear wax
- ☐ Grommet insertion
- ☐ Tympanoplasty
- ☐ Mastoid surgery
- ☐ Hearing aid fitting
- ☐ Cochlear implantation
- ☐ Hearing and speech therapy
- ☐ Educational support for children with hearing loss
- ☐ Others (please specify): ....

Are any outreach services for EHC available at the secondary level?

☐ yes ☐ no

If yes, please give details about the nature of the services, frequency, coordinating authority on a separate sheet

If a person reports with an ear or hearing problem that requires special attention, to where is he or she referred from the secondary level of care?

- ☐ Tertiary hospital
- ☐ Private facility
- ☐ Other (please specify): ....
- ☐ There is no referral

Comments and additional information:

2.2.4 Tertiary level

Are ear and hearing care services available at tertiary level?

☐ yes ☐ no

If yes, what type of facility provides EHC services? ....

What is the health facility at tertiary level providing EHC services? ....

If there are multiple facilities, please attach additional sheets.

What is the population (in 1000s) for which the facility caters? ....

Who are the health care providers at the tertiary level facilities?

(Please tick all service providers involved in health care services at tertiary level facilities.)

- ☐ General physicians
- ☐ ENT Specialists
- ☐ Audiologists
- ☐ Speech therapists/Auditory-Verbal therapists
- ☐ Hearing aid technicians/Ear mould technicians
- ☐ Teachers of the Deaf
- ☐ Sign-language interpreters
- ☐ Paediatricians
- ☐ Obstetricians
- ☐ Others (please specify):

....

....

Which of the following EHC services are provided at this level?

(Please tick all services that are available at the tertiary level facilities.)

- ☐ Health awareness
- ☐ Information, education and communication
- ☐ Ear examination through otoscopy
- ☐ Hearing assessment
- ☐ Treatment of acute otitis media
- ☐ Removal of foreign bodies from ear
- ☐ Removal of ear wax
- ☐ Grommet insertion
- ☐ Tympanoplasty
- ☐ Mastoid surgery
- ☐ Hearing aid fitting
- ☐ Cochlear implantation
- ☐ Hearing and speech therapy
- ☐ Educational support for children with hearing loss
- ☐ Others (please specify): ....

Are outreach services (eg. camps) for EHC available at the tertiary level?

☐ Yes ☐ No

If yes, please give details about the nature of the services, frequency and coordinating authority on a separate sheet

If a person reports with an ear or hearing problem that requires special attention, to where is he or she referred?

....

What percentage of EHC services are provided by:

- the public health system: .... %
- the private sector: .... %

What percentage of health care services are provided by:

- the public health system: .... %
- the private sector: .... %

Comments and additional information:

2.2.5	Community level
	**Are there any programmes at community level that may be relevant to EHC, other than the primary level services mentioned in 2.2.2?**<br>☐ yes ☐ no<br>If yes, please indicate what these programmes are:<br>……………………………………………………………………………………<br>Additional details can be attached, if required.
	**Are any outreach services for EHC available at the community level?**<br>☐ yes ☐ no<br>If yes, please give details of the nature of the services, frequency and coordinating authority on a separate sheet.
2.2.6	**Is there an established referral system across the continuum of care (from primary to secondary to tertiary level)?**<br>☐ yes ☐ no
2.2.7	**What proportion of ear and hearing care services is provided by private hospitals and service providers?**<br>……………………………………………………………………………………
Comments and additional information:	

How would you summarize the overall situation regarding services for provision of EHC in the country?

Circle the most suitable response. If none of the statements accurately reflects the situation in your country, add what seems most appropriate in your country's context.

1	EHC services (including surgical and audiological services) are not available at tertiary, secondary or primary level.
2	EHC services (including surgical and audiological services) are available in limited centres at tertiary level only. There are no EHC services at primary and secondary levels.
3	EHC services (including surgical and audiological services) are available at all tertiary level centres. There are no EHC services at primary and secondary levels.
4	EHC services (including surgical and audiological services) are available at all tertiary and secondary level health facilities. No EHC services are provided at primary level health facilities.
5	EHC services (including surgical and audiological services) are available at all tertiary, secondary and primary level health facilities.
6	None of the above. The current status can be stated as:

2.3 HEALTH WORKFORCE

2.3.1 ENT specialists

Total number in the country: ....

Ratio: .... per 100 000 population

Distribution urban:rural: .... : ....

Minimum educational qualification required: ....

Are there educational institutions offering degree or diploma courses for training of ENT specialists?

☐ yes ☐ no

If yes,

total number of educational facilities offering degree or diploma in ENT: ....

Number of persons graduating each year: ....

If no,

what are the options for training of ENT specialists for the country?

....

Skills of the ENT specialists

☐ Medical management of common ear conditions

Surgical management of common ear conditions

- ☐ myringotomy
- ☐ grommet insertion
- ☐ tympanoplasty
- ☐ mastoid surgery
- ☐ stapedectomy
- ☐ cochlear implantation

☐ Audiometry
☐ Tympanometry
☐ Otoacoustic emissions/Auditory brainstem response/Auditory steady state response
☐ Hearing aid fitting
☐ Ear mould preparation
☐ Audio-verbal therapy
☐ Speech therapy
☐ Others (please specify) ....

Comments and additional information:

2.3.2 Audiologists

Total number in the country: ....

Ratio: .... per 100 000 population

Distribution urban:rural: .... : ....

Minimum educational qualification required: ....

Are there educational institutions offering degree or diploma courses for training of audiologists?
☐ yes ☐ no

If yes,

total number of educational facilities offering degree or diploma in audiology: ....

Number of persons graduating each year: ....

If no,
what are the options for training of audiologists for the country?

....

Skills of audiologists

- ☐ Audiometry
- ☐ Tympanometry
- ☐ Oto-acoustic emissions/Auditory brainstem response/Auditory steady state response
- ☐ Hearing aid fitting
- ☐ Audio-verbal therapy
- ☐ Speech therapy
- ☐ Family counselling
- ☐ Counselling on use of hearing aids
- ☐ Ear mould preparation
- ☐ Use of environmental aids
- ☐ Otoscopy
- ☐ Diagnosis of common ear conditions
- ☐ Others (please specify): ....

Comments and additional information:

2.3.3 Speech therapists, audio-verbal therapists, auditory-oral therapists

Total number in the country: ....

Ratio: .... per 100 000 population

Distribution urban:rural: .... : ....

Minimum educational qualification required: ....

Are there educational institutions offering degree or diploma courses for training of speech therapists or audio-verbal/auditory-oral therapists?

☐ yes ☐ no

If yes,

total number of educational facilities offering degree or diploma course: ....

Number of persons graduating each year: ....

If no,

what are the options for training of speech therapists and audio-verbal therapists for the country?

....

Skills of speech and audio-verbal therapists

- ☐ Audio-verbal therapy
- ☐ Speech therapy
- ☐ Family counselling
- ☐ Counseling on use of hearing aids
- ☐ Audiometry
- ☐ Tympanometry
- ☐ Otoacoustic emissions/Auditory brainstem response/Auditory steady state response
- ☐ Hearing aid fitting
- ☐ Ear mould preparation
- ☐ Use of environmental aids
- ☐ Others (please specify): ....

Comments and additional information:

2.3.4 Hearing aid technicians and ear mould technicians

Total number in the country: ....

Ratio: .... per 100 000 population

Distribution urban:rural: .... : ....

Minimum educational qualification required: ....

Are there educational institutions offering degree or diploma courses for training of hearing aid or ear mould technicians?

☐ yes ☐ no

If yes,

total number of educational facilities offering suitable degree or diploma: ....

Number of persons graduating each year: ....

If no,

what are the options for training of hearing aid and ear mould technicians for the country?

....

Skills of hearing aid and ear mould technicians

- ☐ Hearing aid fitting
- ☐ Ear mould preparation
- ☐ Audio-verbal therapy
- ☐ Speech therapy
- ☐ Family counselling
- ☐ Counselling on use of hearing aids
- ☐ Use of environmental aids
- ☐ Others (please specify): ....

Comments and additional information:

2.3.5 Teachers of the deaf

Total number in the country: ....

Ratio: .... per 100 000 population

Distribution urban:rural: .... : ....

Minimum educational qualification required: ....

Are there educational institutions offering degree or diploma courses for training teachers of the deaf?

☐ yes ☐ no

If yes,

total number of educational facilities offering suitable degree or diploma: .....

Number of persons graduating each year: ....

If no,

what are the options for training of teachers of the deaf for the country?

....

Skills

- ☐ Educational support for preschool children with hearing loss
- ☐ Educational support for schoolchildren with hearing loss
- ☐ Sign language practice
- ☐ Family counselling
- ☐ Counseling on use of hearing aids
- ☐ Use of environmental aids
- ☐ Others (please specify): ....

Comments and additional information:

2.3.6 Sign language interpreters

Total number in the country: ....

Ratio: .... per 100 000 population

Distribution urban:rural: .... : ....

Which governmental and nongovernmental agencies employ sign language interpreters?

....

Minimum educational qualification required: ....

Are there educational institutions offering degree or diploma courses for training of sign language interpreters?

☐ yes ☐ no

Which agency accredits the courses? ....

If yes,

total number of educational facilities offering suitable degree or diploma: ....

Number of persons graduating each year: ....

If no,

what are the options for training of sign language interpreters for the country?

....

Skills

- ☐ Sign language interpretation
- ☐ Family and individual counselling
- ☐ Counseling for use of hearing aids
- ☐ Use of environmental aids
- ☐ Others (please specify): ....

Comments and additional information:

2.3.7 General physicians (engaged through the national health service)

Total number in the country: ....

Ratio: .... per 100 000 population

Distribution urban:rural: .... : ....

Minimum educational qualification required: ....

Which of the following ear and hearing care services do general physicians provide?

- Diagnosis and medical management of acute ear infections: ☐ yes ☐ no
- Diagnosis and medical management of chronic ear infections: ☐ yes ☐ no
- Hearing tests: ☐ yes ☐ no
  If yes, which tests can they perform: ....
- Removal of ear wax: ☐ yes ☐ no
- Removal of foreign bodies: ☐ yes ☐ no
- Any other (please specify) ....

Comments and additional information:

2.3.8 Health workers

(if there is more than one cadre of health workers, include details separately for each).

Total number in the country: ....

Ratio: .... per 100 000 population

Distribution urban:rural: .... : ....

Minimum educational qualification required: ....

Which agency provides training for health workers? ....

Which of the following services do health workers provide?

- Raising health awareness in the community: ☐ yes ☐ no
- Medical management of ear infections: ☐ yes ☐ no
- Provision of ear drops: ☐ yes ☐ no
- Referral services: ☐ yes ☐ no
- Hearing tests: ☐ yes ☐ no

  If yes, which tests can they perform? ....

- Wax removal: ☐ yes ☐ no
- Removal of foreign bodies: ☐ yes ☐ no
- Any other (please specify) ....

Comments and additional information:

2.3.9 Please note below any other cadres of personnel who are providing or can be engaged for the provision of ear and hearing care in the country.

Cadre: ....

Total number: .... **Ratio:** .... per 100 000 population

Training provided by: ....

Duties: ....

....

Cadre: ....

Total number: .... **Ratio:** .... per 100 000 population

Training provided by: ....

Duties: ....

....

How would you summarize the overall situation regarding availability of human resources for ear and hearing care within the country?

Circle the most suitable response. If none of the statements accurately reflects the situation in your country, add what seems most appropriate in your country's context.

1	There is a severe shortage of all levels of human resources required for EHC services in the country.
2	There are an adequate number of ENT specialists available in urban areas but not in rural parts of the country. There is a shortage of other categories of human resources.[5]
3	There are an adequate number of human resources for EHC[6] available in urban areas. This is not so in rural parts of the country.
4	There are an adequate number of human resources for EHC[7] available in all urban areas and some rural areas of the country.
5	There are an adequate number of human resources for EHC[7] available in all urban areas and rural areas of the country.
6	None of the above. The current status can be stated as:

[5] Required for provision of audiological, rehabilitative, educational and support services.
[6] Required for provision of audiological, rehabilitative, educational and support services.
[7] Required for provision of medical, surgical, audiological, rehabilitative, educational and support services.

How would you summarize the overall situation regarding availability of educational facilities for training of human resources for EHC within the country?

Circle the most suitable response. If none of the statements accurately reflects the situation in your country, add what seems most appropriate in your country's context.

1	Educational facilities for development of human resources for EHC are not available in the country.
2	Training facilities for health workers in EHC are available. There are no educational facilities for professional training of ENT specialists, audiologists and other cadres.
3	Training facilities for health workers in EHC are available. Educational facilities for professional training of human resources for hearing care are also available, but these are inadequate to provide EHC for the entire country.
4	Training facilities for health workers are available as well as educational facilities for professional training, and these are adequate to provide EHC for the entire country.
5	None of the above. The current status can be stated as:

2.4 MEDICAL PRODUCTS AND HEALTH TECHNOLOGY

2.4.1 Hearing devices

Hearing aids

Are hearing aid services available through the public health system?

☐ yes ☐ no

If yes,

What types of hearing aid are available through the public health system? (tick as appropriate)

☐ Digital technology ☐ analogue technology ☐ both

☐ Body-worn ☐ behind-the ear ☐ In the ear ☐ both

☐ Open fit ☐ with custom-made ear moulds

Is there standardized guidance or recommendations for hearing aid fitting in

- adults? ☐ Yes ☐ No
- children? ☐ Yes ☐ No

Are hearing aids fitted:

☐ Free of cost ☐ at subsidized cost ☐ at full cost

Are repair and maintenance issues included in the hearing aid fitting programme?

☐ yes ☐ no

Is battery provision part of the hearing aid fitting programme?

☐ yes ☐ no

Are hearing aid services available in the private sector?

☐ yes ☐ no

Are any nongovernmental organizations providing hearing aid services?

☐ yes ☐ no

If yes, please list names: ....

Cochlear implants(CI)

Are CI services available through the public health system?

☐ yes ☐ no

Are CI services available in the private sector?

☐ yes ☐ no

Is there standardized guidance or recommendations on cochlear implantation?

☐ yes ☐ no

Comments and additional information:

2.4.2 Other assistive devices

Loop systems

Are loop systems available in public places?

☐ yes ☐ no

Are loop systems available in schools or special schools?:

☐ yes ☐ no

Captioning services

Are captioning services available on majority of TV channels?

☐ yes ☐ no

Comments and additional information:

2.4.3 Medicines

Which of the following medicines are available through the public health system and at which care level? (tick as applicable)

- Broad spectrum antibiotics: ☐ Not available ☐ primary ☐ secondary ☐ tertiary
- Antibiotic ear drops: ☐ Not available ☐ primary ☐ secondary ☐ tertiary
- Antifungal ear drops: ☐ Not available ☐ primary ☐ secondary ☐ tertiary
- Nasal decongestant: ☐ Not available ☐ primary ☐ secondary ☐ tertiary

Comments and additional information:

How would you summarize the overall situation regarding the availability and accessibility hearing devices in the country?

Circle the most suitable response. If none of the statements accurately reflects the situation in your country, add what seems most appropriate in your country's context.

1	There is no availability of hearing aid services or cochlear implantation in the country.
2	Hearing aid services[8] are available but are not accessible to most (because of high cost or location of services). CI[9] are not accessible to the majority.
3	Hearing aid services[8] are available and accessible to most people in urban areas of the country. However, CI[9] are not accessible to the majority.
4	Hearing aid services[8] are available and accessible to most people in urban and rural parts of the country. However, CI[9] are not accessible to the majority.
5	Hearing aid services are available and accessible to most people in urban and rural areas of the country. CI are available and accessible to those requiring them.
6	None of the above. The current status can be stated as:

[8] Including fitting and maintenance.
[9] Including follow-up and therapy.

2.5 HEALTH FINANCING

2.5.1 Financing of ear and hearing care services

Is there a budget allocation for EHC in the Ministry of Health? ☐ yes ☐ no

If yes, what is the annual allocation? ....

Does the government provide EHC services? ☐ yes ☐ no

If yes, select the most suitable response below:

☐ All services are available free of charge.
☐ A nominal fee has to be paid by patients.
☐ Some (EHC) services are available free of charge but others have to be paid for, fully or partially.
☐ Costs are fully covered by health insurance.
☐ Costs are partially covered by health insurance.
☐ Costs are borne completely by patients.

Are EHC services provided by privately practicing specialists? ☐ yes ☐ no

If yes, select the most suitable response below:

☐ Costs are fully covered by health insurance
☐ Costs are partially covered by health insurance
☐ Costs are borne completely by patients

Are EHC services provided by any nongovernmental agency? ☐ yes ☐ no

If yes, select the most suitable response below:

☐ EHC services are completely free
☐ Some services are available free of charge while others have to be paid for, fully or partially.
☐ There is a nominal charge for EHC services.

Comments and additional information:

2.5.2 Health insurance

Is health insurance available in the country?

If yes, what percentage of the population is covered by it? .... %

Who are the key providers of health insurance in the country?

☐ Government
☐ Employers
☐ Private insurance companies (give names of companies)

....

....

....

Comments and additional information:

How would you summarize the overall situation regarding the health financing in the country?

Circle the most suitable response. If none of the statements accurately reflect the situation in your country, add what seems most appropriate in your country's context.

1	EHC services are not affordable and no health financing is available.
2	Part of the expenses for EHC are covered through government-led health financing schemes, but it is still not affordable for the majority.
3	Most of the expenses for EHC are covered through government-led health financing schemes, and it is affordable for the majority.
4	Most of the expenses for EHC are covered through government-led health financing schemes, and it is affordable for all.
5	All expenses related to EHC are covered through government-led health financing schemes.
6	None of the above. The current status can be stated as:

2.5 HEALTH FINANCING

2.5.1 Financing of ear and hearing care services

Is there a budget allocation for EHC in the Ministry of Health? ☐ yes ☐ no

If yes, what is the annual allocation? ....

Does the government provide EHC services? ☐ yes ☐ no

If yes, select the most suitable response below:

- ☐ All services are available free of cost.
- ☐ A nominal fee has to be paid by patients.
- ☐ Some services are available free of charge but others have to be paid for, fully or partially.
- ☐ Costs are fully covered by health insurance.
- ☐ Costs are partially covered by health insurance.
- ☐ Costs are borne completely by patients.

Are EHC services provided by privately practising specialists? ☐ yes ☐ no

If yes, select the most suitable response below:

- ☐ Costs are fully covered by health insurance.
- ☐ Costs are partially covered by health insurance.
- ☐ Costs are borne completely by patients.

Are EHC services provided by any nongovernmental agency? ☐ yes ☐ no

If yes, select the most suitable response below:

- ☐ EHC services are completely free.
- ☐ Some services are available free of charge while others have to be paid for, fully or partially.
- ☐ There is a nominal charge for EHC services.

Comments and additional information:

2.5.2 Health insurance

Is health insurance available in the country?

If yes, what percentage of the population is covered by it? .... %

Who are the key providers of health insurance in the country?

- ☐ Government
- ☐ Employers
- ☐ Private insurance companies (give names of companies)

....

....

....

Comments and additional information:

How would you summarize the overall situation regarding health financing in the country? Circle the most suitable response. If none of the statements accurately reflects the situation in your country, add what seems most appropriate in your country's context.	
1	EHC services are not affordable and no health financing is available.
2	Part of the cost of EHC is covered through government-led health financing schemes, but it is still not affordable for the majority.
3	Most of the cost of EHC is covered through government-led health financing schemes, and it is affordable for the majority.
4	Most of the cost of EHC is covered through government-led health financing schemes, and it is affordable for all.
5	All the costs of EHC are covered through government-led health financing schemes.
6	None of the above. The current status can be stated as:

2.6 HEALTH INFORMATION AND RESEARCH

2.6.1 Health information system

Health-related data

Does the country have a national health information system?
☐ yes ☐ no

Are health related data and information collected and centrally administered in the country?
☐ yes ☐ no

If yes, which agencies are responsible for data collection, analysis and reporting?

....

If no, is there any other mechanism for obtaining health-related information in the country?

....

What are the sources of health information reported by the country?

☐ Programme reports
☐ Hospital data
☐ Census data
☐ Data from surveys

Is there a system of personal child health records (through health or other departments)?
☐ yes ☐ no

If yes, which agency or department is responsible for developing recording tools (such as child health cards) and monitoring the records? ....

Is information on ear or hearing included as part of this record?
☐ yes ☐ no

Is any information or indicator related to EHC reported at government level?
☐ yes ☐ no

If yes, please list the information and indicate the source.

....

....

....

Comments and additional information:

2.6.2 Research

Are there any government-led agencies or institutes conducting research in the field of EHC?

☐ yes ☐ no

If yes, give the names of the agencies and institutes:

....

....

What are the key areas of focus of the research in the field of EHC?

- ☐ Epidemiology
- ☐ Clinical
- ☐ Diagnostics
- ☐ Operational
- ☐ Hearing devices
- ☐ Others (please specify)

....

....

Which are the key funding agencies supporting research in the field of EHC?

....

....

Comments and additional information:

How would you summarize the overall situation regarding the health information system in the country?

Circle the most suitable response. If none of the statements accurately reflects the situation in your country, add what seems most appropriate in your country's context.

1	There is no functional health information system in the country.
2	There is no government-led health information system in the country.<br>Health-related indicators are collated by state or provincial governments or NGOs.
3	There is a government-led health information system in the country.<br>It does not include information or indicators on EHC.
4	There is a government-led health information system in the country.<br>It includes some information or indicators on EHC.
5	There is a government-led health information system in the country.<br>It includes all relevant information and indicators on EHC.
6	None of the above. The current status can be stated as:

SECTION 3: STAKEHOLDER ANALYSIS

Please mention all potential and existing stakeholders in your country that are or could be involved in ear and hearing care services. Identify the lead or responsible position or person, where possible, and give contact details.

**3.1**	**Departments in Ministry of Health** (list in order of perceived priority for collaboration in developing and implementing EHC strategy)		
	Name of department	Lead/contact person (name and designation)	Contact details
1.			Address: ...<br>Telephone numbers: ...<br>Email: ...
2.			Address: ...<br>Telephone numbers: ...<br>Email: ...
3.			Address: ...<br>Telephone numbers: ...<br>Email: ...
4.			Address: ...<br>Telephone numbers: ...<br>Email: ...
5.			Address: ...<br>Telephone numbers: ...<br>Email: ...
6.			Address: ...<br>Telephone numbers: ...<br>Email: ...

3.2	**Other ministeries and departments**<br>(list in order of perceived priority for collaboration in developing and implementing EHC strategy)		
	Name of ministry or department	Lead/contact person (name and designation)	Contact details
1.			Address: ....<br>Telephone numbers: ....<br>Email: ....
2.			Address: ....<br>Telephone numbers: ....<br>Email: ....
3.			Address: ....<br>Telephone numbers: ....<br>Email: ....
4.			Address: ....<br>Telephone numbers: ....<br>Email: ....
5.			Address: ....<br>Telephone numbers: ....<br>Email: ....
6.			Address: ....<br>Telephone numbers: ....<br>Email: ....

3.3	**Professionals and academics**<br>(list in order of experience and interest in, the development and implementation of EHC strategy)		
	Name of institute or association	Lead/contact person (Name and designation)	Contact details
1.			Address: ……………<br>Telephone numbers: ……………<br>Email: ……………
2.			Address: ……………<br>Telephone numbers: ……………<br>Email: ……………
3.			Address: ……………<br>Telephone numbers: ……………<br>Email: ……………
4.			Address: ……………<br>Telephone numbers: ……………<br>Email: ……………
5.			Address: ……………<br>Telephone numbers: ……………<br>Email: ……………
6.			Address: ……………<br>Telephone numbers: ……………<br>Email: ……………

3.4	**Civil society groups, including disabled persons' organizations** (list in order of perceived priority for collaboration in developing and implementing EHC strategy)		
	Name of group or organization	Lead/contact person (name and designation)	Contact details
1.			Address: ...<br>Telephone numbers: ...<br>Email: ...
2.			Address: ...<br>Telephone numbers: ...<br>Email: ...
3.			Address: ...<br>Telephone numbers: ...<br>Email: ...
4.			Address: ...<br>Telephone numbers: ...<br>Email: ...
5.			Address: ...<br>Telephone numbers: ...<br>Email: ...
6.			Address: ...<br>Telephone numbers: ...<br>Email: ...

3.5 UN agencies

	Name of agency	Lead/contact person (Name and designation)	Contact details
1.			Address: ....<br>Telephone numbers: ....<br>Email: ....
2.			Address: ....<br>Telephone numbers: ....<br>Email: ....
3.			Address: ....<br>Telephone numbers: ....<br>Email: ....
4.			Address: ....<br>Telephone numbers: ....<br>Email: ....

3.6	**Industry partners**<br>(list in order of perceived priority, based on need for collaboration and possible interest of the stakeholder, in developing and implementing EHC strategy)		
	Name of company	Lead/contact person (name and designation)	Contact details
1.			Address: ....<br>Telephone numbers: ....<br>Email: ....
2.			Address: ....<br>Telephone numbers: ....<br>Email: ....
3.			Address: ....<br>Telephone numbers: ....<br>Email: ....
4.			Address: ....<br>Telephone numbers: ....<br>Email: ....
5.			Address: ....<br>Telephone numbers: ....<br>Email: ....
6.			Address: ....<br>Telephone numbers: ....<br>Email: ....

3.7	**Financial sponsors**<br>(list in order of perceived priority, based on existing operations in the field of EHC and possible interest and in developing and implementing EHC strategy)		
	Name of organization	Lead/contact person (name and designation)	Contact details
1.			Address: ……<br>Telephone numbers: ……<br>Email: ……
2.			Address: ……<br>Telephone numbers: ……<br>Email: ……
3.			Address: ……<br>Telephone numbers: ……<br>Email: ……
4.			Address: ……<br>Telephone numbers: ……<br>Email: ……
5.			Address: ……<br>Telephone numbers: ……<br>Email: ……
6.			Address: ……<br>Telephone numbers: ……<br>Email: ……

3.8	Any other groups, individuals or organizations		
	Name of organization	Lead/contact person (name and designation)	Contact details
1.			Address: ......<br>Telephone numbers: ......<br>Email: ......
2.			Address: ......<br>Telephone numbers: ......<br>Email: ......
3.			Address: ......<br>Telephone numbers: ......<br>Email: ......
4.			Address: ......<br>Telephone numbers: ......<br>Email: ......
5.			Address: ......<br>Telephone numbers: ......<br>Email: ......
6.			Address: ......<br>Telephone numbers: ......<br>Email: ......

How would you summarize the overall situation regarding the interest and commitment of stakeholders to EHC?

Circle the most suitable response. If none of the statements accurately reflects the situation in your country, add what seems most appropriate in your country's context.

1	There are currently no interested stakeholders in governmental and nongovernmental sectors for promotion of EHC.
2	A few stakeholders are committed to EHC. This is insufficient to drive forward the policy development and implementation process.
3	Multiple groups of stakeholders, including government, are committed to EHC. However, there is a lack of stakeholders that can provide financial support to drive the process.
4	Multiple groups of stakeholders are committed to EHC, including government and financial sponsors.
5	All groups of stakeholders are committed to EHC, including financial sponsors.
6	None of the above. The current status can be stated as:

SECTION 4: LIST OF SOURCES

Please provide a list of sources used in this situation analysis.

1	**Resource persons** (name and designation)	**Contact details** (telephone number and email)

2	**Literature reviewed** (peer reviewed articles, books, online resources, any other)

SUGGESTED REPORTING FORMAT

Further guidance on using the situation analysis results is provided elsewhere.[10]

Part 1	On the basis of the information obtained, describe the key points relevant to EHC strategy development in the country.
1.	Need for ear and hearing care (EHC) services
2.	Leadership for EHC and governance structure
3.	Current service delivery for EHC services
4.	Availability of health workforce for EHC service provision and educational opportunities
5.	Availability and accessibility of medical products and health technology
6.	Inclusion of ear and hearing in health information system and focus of ongoing research
7.	Availability and potential interest of stakeholders in promoting EHC strategies

[10] Ear and hearing care: planning and monitoring of national strategies. A manual. Geneva, World Health Organization, 2015.

**Part 2**	**On the basis of the information obtained and the responses to the summary tables, summarize the current situation regarding EHC in the country. If desired, you may add comments on the targets to be achieved in the next 5 years.**

	1	2	3	4	5	6 (Options 1–5 not applicable)	Comments
Preparedness for EHC strategy							
Leadership and governance							
Service delivery							
Health workforce							
Capacity for education and training							
Technology							
Health financing							
Health information							
Stakeholder participation							

**Part 3**	**Additional Comments:**

Part 3	Next steps and key recommendations
1.	
2.	
3.	
4.	
5.	
6.	

ANNEX 1
GUIDANCE FOR USE

This annex provides guidance on how to complete the questionnaire and on resources that may provide needed information. The suggested sources are not exhaustive, and are not always needed. The guidance may be referred to as and when required.

The summary tables at the end of each section of the questionnaire give a simple overview of the current situation. The statements in the tables can also serve as possible targets when an ear and hearing care strategy is being developed.

1.1–1.3 Population profile

These sections require detailed information on the population, sociodemographic information and health status. Possible resources include the following.

- National census data
- UN Department of Economic and Social Affairs
- World Bank
- WHO immunization monitoring and surveillance data
- Global Health Observatory
- WHO State of inequality report
- WHO tuberculosis country profile
- UNAIDS

1.4 Hearing loss

Disabling hearing loss is defined as a hearing threshold greater than 40dB in the better ear in adults, greater than 30dB in the better ear in children (http://www.who.int/pbd/deafness/estimates/en/).

In section 1.4.2, the age distribution may be given in terms of percentage of population or percentage of those with hearing loss. The choice should be indicated.

In section 1.4.4, the main causes of hearing loss in the country should be ranked in order of their prevalence or contribution to burden of disease. The ranking should preferably be based on epidemiological studies. Where no such studies are available, the ranking may be based on input from hearing care professionals. Ideally, measures of hearing loss should refer to permanent hearing loss.

1.5 Health care strategy

Information about public health policies may be available from the Ministry of Health and can be obtained through interviews with relevant personnel. Such information may also be found in public health publications or on the Ministry websites.

2	Assessment of health system capacity
	The information for this section is likely to come from a limited number of sources, such as the Ministry of Health. It would be helpful to identify sources in the Ministry of Health who can provide information on the health care delivery infrastructure and the status of any ear and hearing care strategy. Documents, publications and websites of the Ministry of Health may also provide this information.<br><br>In addition, it may be useful to discuss with experts in the field of public health, working either for the Ministry or at leading universities. The information obtained can be supplemented through discussions with ear and hearing care professionals working in the public health sector.<br><br>You should include as much detail as possible in the sections that request additional details.
**2.1**	**Leadership and governance**
2.1.1	**Sources: see above (section 2)**<br><br>A national committee is a formal body appointed by the government to assess the problem of ear diseases and hearing loss in the country or to develop suitable plans for their reduction.
2.1.2	A strategic plan or policy refers to a concept paper, strategic plan or similar for prevention or reduction of ear diseases or hearing loss in all or part of the country thereof. Include as much detail as is available, e.g. on duration, components, implementation, coverage (geographical, population, age groups, etc.), private-public partnerships.
2.1.3	Programmes being implemented by the government may be vertical, stand-alone programmes or may be part of a broader activity. For example, hearing screening may be undertaken in schools as a separate activity mandated or organized by the government (government-led school hearing screening programme). Alternatively, hearing screening may be carried out in schools as part of a broader health check-up among the children. Both aspects should be explored and asked about. The same is true for neonatal and infant hearing screening and provision of hearing devices.<br><br>In relation to hearing devices provided under the programme, these may include hearing aids, cochlear implants, bone anchored hearing aid and any other devices used for improved hearing.

2.2	Service delivery
	This section seeks information about health care facilities and providers at the different levels of service delivery. The purpose is to understand which health facilities and service providers are, or can be, engaged in delivering ear and hearing care services. This information will be useful when services and training are being planned as part of an ear and hearing care strategy. The section can best be filled in after consultation with public health experts who have knowledge and information about the country's health care infrastructure and service providers. The availability of services and human resources may vary from one part of the country to another. In that case, the minimum standards or practices mandated by the federal or central government should be reported.
2.2.2	**Sources: see above**<br><br>Primary care is delivered by health professionals who act as a first point of consultation for all patients in the health care system. Where required, additional information may be provided on a separate sheet.
2.2.3	Secondary care is provided by medical specialists and other health professionals who generally do not have first contact with patients, for example, cardiologists, urologists and dermatologists. Where required, additional information may be provided on a separate sheet.
2.2.4	Tertiary care is specialized consultative health care, usually for inpatients and on referral from a health professional at primary or secondary level, in a facility that has the personnel and facilities to carry out advanced medical investigations and treatment, such as a tertiary referral hospital. Where required, additional information may be provided on a separate sheet.
2.2.5	A community-level programme is care delivered in the community, within the governmental structure but not included as part of the primary care package described in 2.2.2. Where required, additional information may be provided on a separate sheet.

2.3	Health workforce
	**Sources: see above**<br><br>Sources will vary between countries; some information may be available through government departments, while information about specialized personnel, such as otolaryngologists and audiologists, may be obtained from their respective professional organizations. Information on regulated professions may be available through the regulator; examples of regulators include the Rehabilitation Council of India, and the Health and Care Professions Council and ENTUK in the United Kingdom.
2.3.1	Specialists are medical doctors who have received training in surgery of the ear, nose and throat through a formal degree or diploma course.
2.3.2	An audiologist is a person who has obtained a formal degree or diploma in audiology.
2.3.3	Speech therapist/auditory-verbal therapist/auditory-oral therapist refers to a person trained (through a formal diploma or degree programme) to assist children with hearing loss to communicate using spoken language.
2.3.4	Hearing aid technicians and ear mould technicians have received training in the production, maintenance, and fitting of hearing aids.
2.3.5	Teachers of the deaf have undergone formal training and received a certificate, diploma or degree in teaching of persons with hearing impairment.
2.3.6	Sign language interpreters communicate the meaning of spoken or signed language into another spoken or signed language.
2.3.7	General physicians are medical doctors who provide care to patients with acute or chronic illness, provide preventive care, and engage in health education with patients.
2.3.8	Health workers are all people engaged in actions whose primary intent is to enhance health.[11]
2.3.9	Other cadres may include service providers engaged in providing ear and hearing care, such as ENT nurses and audiometricians. This category should also include service providers who can potentially be trained to provide hearing care services, such as community-based rehabilitation workers and traditional birth attendants.
**2.4**	**Medical products and health technology**
	In addition to the sources mentioned above, information about availability of diagnostic tests, devices and medicines may be obtained from industry partners and associations of people who are deaf or hard of hearing.
**2.5**	**Health financing**
	**Sources: see above**

[11] Working together for health: the World Health Report 2006. Geneva, World Health Organization, 2006.

2.6	Health information and research
2.6.1	In addition to the sources mentioned above, information should also be sought from researchers working in the field.<br><br>• Programme reports refer to reporting on specific programmes run by the government, e.g. maternal and child health programme.<br><br>• Hospital data include information such as number of patients seen, morbidity and mortality data, and number and type of interventions.
2.6.2	• Epidemiological research is the study of the distribution and determinants of health-related states or events (including disease), and the application of this study to the control of diseases and other health problems<br><br>• Clinical research is research conducted on human subjects (or on material of human origin, such as tissues, specimens and cognitive phenomena) that involves direct interaction with the subjects.<br><br>• Research in diagnostics refers to research on techniques for identification and diagnosis of hearing loss.<br><br>• Operational research aims to identify solutions to problems that limit programme quality, efficiency or effectiveness, or to determine alternative service delivery strategies for improved outcomes.[12]<br><br>• Research on hearing devices refers to efforts to develop better and more appropriate devices to improve hearing.
**3**	**Stakeholder analysis**
	Information on stakeholders can be obtained through discussion with:<br>• the Ministry of Health;<br><br>• nongovernmental organizations and disabled persons' organizations;<br><br>• ear and hearing care professionals;<br><br>• public health professionals<br><br>Organizations and individuals involved in providing EHC can also be identified through targeted desk-based research.
**4**	**Sources**
	All sources used in the situation analysis, including people interviewed and literature consulted, should be listed.

[12] Guide to operational research in programs supported by the Global Fund. Geneva, World Health Organization and the Global Fund to Fight AIDS, Tuberculosis and Malaria (http://www.who.int/hiv/pub/operational/or_guide_gf.pdf).

Reporting format

The suggested reporting format can be used to summarize the situation analysis and make a report that can be used in planning ear and hearing care strategies.

Part 1. The main points of each section of the questionnaire can be noted here. It is advisable to focus on the aspects most likely to be relevant for the strategic planning process for the country.[13]

Part 2 gives an overview of the responses in the summary tables in each section. This can give an overall idea of the situation in the country and can also be used to indicate the goals that the country would like to set in different sectors over a period of time (say, 5 years). The tables can be used during the planning phase and may help in determining areas of maximum need and hence prioritization. The tables may also help in monitoring changes in provision of ear and hearing care services over time.

Part 4. Suggested next steps or recommendations may be appropriate and can help to provide direction for the development of the national or subnational ear and hearing care strategy.

[13] Ear and hearing care: planning and monitoring national strategies. A manual. Geneva, World Health Organization, 2015.

**FOR MORE INFORMATION,
PLEASE CONTACT:**

Department for Management
of NCDs, Disability, Violence and
Injury Prevention

World Health Organization
Avenue Appia 20
CH-1211 Geneva 27
Switzerland

9 789241 509954
